# Altered Ca^2+^ homeostasis induces Calpain-Cathepsin axis activation in sporadic Creutzfeldt-Jakob disease

**DOI:** 10.1186/s40478-017-0431-y

**Published:** 2017-04-27

**Authors:** Franc Llorens, Katrin Thüne, Beata Sikorska, Matthias Schmitz, Waqas Tahir, Natalia Fernández-Borges, Maria Cramm, Nadine Gotzmann, Margarita Carmona, Nathalie Streichenberger, Uwe Michel, Saima Zafar, Anna-Lena Schuetz, Ashish Rajput, Olivier Andréoletti, Stefan Bonn, Andre Fischer, Pawel P. Liberski, Juan Maria Torres, Isidre Ferrer, Inga Zerr

**Affiliations:** 1 Department of Neurology, University Medical Center Göttingen, and German Center for Neurodegenerative Diseases (DZNE), Robert Koch Strasse 40, 37075 Göttingen, Germany; 2 German Center for Neurodegenerative Diseases (DZNE), Translational Studies and Biomarkers, Site Göttingen, Germany; 3 CIBERNED (Network center for biomedical research of neurodegenerative diseases), Institute Carlos III, Ministry of Health, Madrid, Spain; 4 Department of Molecular Pathology and Neuropathology, Medical University of Lodz, Lodz, Poland; 5 Centro de Investigación en Sanidad Animal (CISA-INIA), Madrid, Spain; 6 Institute of Neuropathology, IDIBELL-University Hospital Bellvitge, University of Barcelona, Hospitalet de Llobregat, Spain; 7 Prion Disease Laboratory, Pathology and Biochemistry, Groupement Hospitalier Est, Hospices Civils de Lyon/Claude Bernard University, Lyon, France; 8 Institut NeuroMyogène CNRS UMR 5310 - INSERM U1217, Lyon, France; 9 German Center for Neurodegenerative Diseases (DZNE), Computational Systems Biology, Site Göttingen, Germany; 10 Institut National de la Recherche Agronomique/Ecole Nationale Vétérinaire, Toulouse, France; 11 German Center for Neurodegenerative Diseases (DZNE), Epigenetics and Systems Medicine in Neurodegenerative Diseases, Site Göttingen, Germany

**Keywords:** Creutzfeldt-Jakob disease, Prion protein, Calpain, Cathepsin, Calcium, Ca^2+^

## Abstract

Sporadic Creutzfeldt-Jakob disease (sCJD) is the most prevalent form of human prion disease and it is characterized by the presence of neuronal loss, spongiform degeneration, chronic inflammation and the accumulation of misfolded and pathogenic prion protein (PrP^Sc^). The molecular mechanisms underlying these alterations are largely unknown, but the presence of intracellular neuronal calcium (Ca^2+^) overload, a general feature in models of prion diseases, is suggested to play a key role in prion pathogenesis.

Here we describe the presence of massive regulation of Ca^2+^ responsive genes in sCJD brain tissue, accompanied by two Ca^2+^-dependent processes: endoplasmic reticulum stress and the activation of the cysteine proteases Calpains 1/2. Pathogenic Calpain proteins activation in sCJD is linked to the cleavage of their cellular substrates, impaired autophagy and lysosomal damage, which is partially reversed by Calpain inhibition in a cellular prion model. Additionally, Calpain 1 treatment enhances seeding activity of PrP^Sc^ in a prion conversion assay. Neuronal lysosomal impairment caused by Calpain over activation leads to the release of the lysosomal protease Cathepsin S that in sCJD mainly localises in axons, although massive Cathepsin S overexpression is detected in microglial cells. Alterations in Ca^2+^ homeostasis and activation of Calpain-Cathepsin axis already occur at pre-clinical stages of the disease as detected in a humanized sCJD mouse model.

Altogether our work indicates that unbalanced Calpain-Cathepsin activation is a relevant contributor to the pathogenesis of sCJD at multiple molecular levels and a potential target for therapeutic intervention.

**Electronic supplementary material:**

The online version of this article (doi:10.1186/s40478-017-0431-y) contains supplementary material, which is available to authorized users.

## Introduction

Prion diseases are a large group of fatal rapid progressive neurodegenerative diseases affecting both humans and animals. The underlying mechanism leading to prion pathogenesis is the conversion of the cellular prion protein (PrP^C^) into the abnormal disease-related form (PrP^Sc^), which accumulates in brain in form of insoluble neurotoxic fibrous structures and amyloid plaques [2, 19]. Prion diseases are categorized according to their etiology in sporadic, familial and iatrogenic forms. Sporadic Creutzfeldt-Jakob disease (sCJD) accounts for about 85% of all prion disease cases, it develops on patients without any known risk factor or gene mutations [77] and it is clinically characterized by the presence of dementia and a broad range of pyramidal, extrapyramidal and cerebellar signs [33, 89].

The combination of codon usage at position 129 of the prion protein gene (*PRNP*) (Methionine or Valine) and the electrophoretic mobility of PrP^Sc^ after Proteinase K (PK) treatment (type 1 or 2), gives raise to different sCJD molecular subtypes characterized by the presence of specific clinicopathological features [31, 73]. sCJD MM1 and VV2 are the most prevalent subtypes with more prominent cortical and cerebellar affection, respectively.

Multiple cellular pathways have been described to be altered in sCJD patients including neuroinflammation, mitochondria function, protein synthesis machinery, purine metabolism, endosomal-lysosomal system and synaptic transmission, among others [4, 56, 59, 62]. In addition, presence of endoplasmic reticulum (ER) and oxidative stress [29, 71, 90, 95] as well as apoptotic mechanisms [27, 37, 55] is well-described in sCJD brain tissue and in models of prion diseases. While it has not been possible to ascertain the main molecular mechanism/s contributing to the neurodegenerative process associated with prion protein pathological conversion, the massive imbalance of physiological functions could explain the rapid progress of sCJD, which is usually fatal within a few months of symptom onset. Thus, neuronal impairment and associated neurodegenerative events are likely to result from the combined and synergic deregulation of multiple physiological processes and functions [54]. Therefore, studies aimed to unveil the very early and upstream events that unchain the cascade of pathological mechanisms are necessary. In this regard, the recent development of humanized sCJD mouse models recapitulating pathological signatures occurring in humans have been demonstrated to be useful in the analysis of the pre-clinical aspects of sCJD pathology [62, 70].

A recurrent observation in prion disease models is the presence of abnormally raised levels of cytosolic Ca^2+^. Prion protein misfolding is one of the contributors to ER stress, resulting in a fast release of Ca^2+^ from intracellular stores to the cytoplasm, an effect that is coupled to the activation of the Unfolded Protein Response (UPR) [46, 65, 90]. Indeed, several evidences point out for a key role of ER stress and UPR activation in the occurrence of synaptic dysfunction and neurodegeneration [91, 92], as well as in the facilitation of prion spreading [44]. In addition, the presence of abnormal or non-functional PrP in the neuronal plasma membrane increases the permeability to physiological ions [60] and modulates the expression and function of Ca^2+^ channels [57, 80]. Sustained cytoplasmic Ca^2+^ elevation is associated with loss of mitochondrial membrane potential, apoptotic and necrotic death, and to the pathogenic activation of the non-lysosomal cysteine proteases Calpains [47]. Pathogenic Calpain activation has been implicated in normal aging as well as in several acute neurological and neurodegenerative conditions, involving abnormal Ca^2+^ influx [6, 18, 86, 98].In Alzheimer’s disease (AD) brain, increased Calpain activation is widely reported [79, 93] and immunoreactivity has been detected in senile plaques [83] and neurofibrillary tangles [39]. Calpain-mediated disruption of lysosomal membrane integrity leads to the leakage of lysosomal Cathepsin proteases, forming the basis of the so-called Calpain-Cathepsin axis hypothesis [103]. Consequently, over activated Calpains and Cathepsins lead to the proteolysis of target and non-target cytoskeletal, cytosolic and nuclear proteins and irreversible cellular damage that ultimately leads to neuronal death [17, 88, 103]. Although evidence suggests the existence of increased Calpain and Cathepsin S expression in scrapie mice [22, 41], final proof of a pathological Calpain-Cathepsin axis activation in prion diseases is lacking.

Here, we present unambiguous evidence for an altered Ca^2+^homeostasis in sCJD brain tissue and propose the existence of the ‘Calpain-Cathepsin’ hypothesis, where Ca^2+^-mediated activation of Calpains results in rupture of lysosomes and leakage of killer Cathepsin S. These mechanisms may act as multifaceted synergistic contributors to the early pathological events of the disease through unregulated cleavage of cellular substrates and organelles and to increase of the seeding activity of pathogenic PrP.

## Material and methods

### Reagents and antibodies

Anti-Ca^2+^/calmodulin-dependent protein kinase II (CamKII)α and anti-CamKIIβ were from Zymed. Anti-phospholipase C (PLC)ε, anti-Protein deglycase DJ-1 (DJ-1), anti-Cathepsin D, anti-B-cell lymphoma 2 (Bcl-2), anti-BCL2 Associated X Protein (Bax), anti-Fas Cell Surface Death Receptor (Fas), anti-Lysosomal associated membrane protein 2 (Lamp2) (H4B4), anti-CCAAT-enhancer-binding protein homologous protein (CHOP/GADD153), were from Santa Cruz. Anti-PLCγ was from Neomarkers. Anti-S100A6, anti-Neurofilament Light (NFL), anti-γ-tubulin and anti-β-actin were from Sigma. Anti-Glyceraldehyde-3-Phosphate Dehydrogenase (GAPDH), anti-Autophagy protein 5(ATG5), anti-Activating transcription factor (ATF)4, anti-Glucose-regulated protein, 78kDa (grp78), anti-X-box binding protein 1(XBP1), anti-Fodrin, anti-calpastatin and anti-calsequestrin 1 were from Abcam. Anti-inositol-requiring enzyme 1 (IRE)1 and anti-microtubule-associated proteins 1A/1B light chain 3A (LC3) were from Cell Signalling. Anti-Cystatin C, anti-CD68 (PG-M1) and anti-HLA-DR, (TAL.1B5) were from Dako. Anti-p-IRE-1 (Ser724), anti-Calpain 1, anti-Calpain 2 and anti-Cathepsin S were from Thermo Fisher. Anti-ATF6 was from Enzo Life Sciences. Anti-Brain-derived neurotrophic factor (BDNF) was from Chemicon. Anti-hsp27 was from Stressgene. Anti-PrP (SAF70) was from Spi-Bio, anti-PrP (3F4) was from Millipore and anti-PrP (SAF32) was from Cayman. Anti-Calpain 1 RP1 (N-ter) was from Triple Point Biologics. Anti-phosphorylated neurofilaments (SMI-31), and anti-neurofilament H non-phosphorylated (SMI-32) were from Covance.

Propidium Iodide was from Sigma, Calpain 1–2 activation kit was from Millipore. Prion protein peptide 106–126 was from JPT Peptides. MDL28170 was from was from ENZO Life Sciences.

### Human cases

Brain tissue was obtained from the Institute of Neuropathology Brain Bank (HUB-ICO-IDIBELL Biobank) and the Biobank of Hospital Clinic-IDIBAPS following the guidelines on this matter of both Spanish legislation and the local ethics committee. Neuropathological examination and characterization was carried out in every case on paraffin-embedded samples. Detailed information of neuropathology, inflammatory profiling and demographics of the sCJD cohort is described as previously [61–63]. sCJD MM1 and VV2 cases were selected due to their higher prevalence but different clinical phenotypes [31, 73]. The presence of infectious, metabolic and neoplastic diseases was discarded in control samples. No correlation between post-mortem delay or sample storage time and levels of proteins and mRNA analysed was observed.

### sCJD MM1 mice–tg340-*PRNP*129MM

The tg340 mouse line expressing about 4-fold level of human PrP M129 on a mouse PrP null background was generated as described before [70]. Control or sCJD MM1 brain tissues as 10% (w/v) homogenates were inoculated in 6–10 week-old mice in the right parietal lobe using a 25-gauge disposable hypodermic needle. Mice were observed daily and the neurological status was assessed weekly. The animals were sacrificed at pre-symptomatic (pre-clinical: 120 dpi) and symptomatic (early clinical: 160 dpi and late clinical: 183 dpi) stages. Additionally, sCJD MM1 inoculum dilutions were performed to study prolonged disease times; animals were sacrificed at 210 dpi (10–1 dilution). A part of the brain was fixed by immersion in 10% buffered formalin to quantify spongiform degeneration and perform immunohistological procedures. The other part was frozen at −80°C to extract protein and RNA. Survival time was calculated for each isolate and expressed as the mean of the survival day post-inoculation (dpi) of all mice scoring positive for PrP^Sc^. Infection rate was determined as the proportion of mice scoring positive for PrP^Sc^ from all inoculated mice. Every effort was made to minimize detrimental effects on animals.

### Primary cell cultures and treatments

For preparation of cortical neurons, pregnant Wistar rats were killed by CO_2_-inhalation at embryonic day 18. The brain from the embryos was taken and the cortex was cleaned from surrounding brain tissue. The remaining cortex was subsequently trypsinized with 1 ml 0.25% Trypsin/EDTA (Gibco) for 12 min at 37°C. 50 μl DNase I (Roche) was added and the tissue was dissociated using a fire-polished Pasteur pipette. Cells were seeded on poly-L-ornithine/laminin (Sigma)-coated glass cover slips or directly on poly-L-ornithine/laminin-coated 24-well culture plates in a density of 350.000 cells per well. Cultures were maintained at 37 °C in a humidified atmosphere at 5% CO2. Culture medium was based on neurobasal medium (Gibco) containing additional transferrin (Applichem), penicillin/streptomycin/neomycin (PSN) (Gibco), L-glutamine (Sigma), and B27 supplement (Gibco). At day in vitro (DiV) 7, cells were treated with 5 μM MDL28170, when indicated, 1h before prion protein peptide treatment (100 μM). Prion protein peptide was prepared as reported before [13]. As control treatment cells were incubated with equal concentration of non-aggregated prion protein peptide. After 24 h of prion protein peptide treatment, cells were analysed with Lysotracker (ThermoFisher) following the manufacturer’s instructions. Calpain activity (Calpain assay kit – Millipore), fixed with PBS-PFA 4% for immunohistochemistry or collected for western-blot experiments. At 48h post-treatment cell viability was analysed with Propidium Iodide.

### Western-blot

Tissues were lysed in Lysis Buffer containing: 100 mM Tris pH 7, 100 mM NaCl, 10 mM EDTA, 0.5% NP-40 and 0.5% Sodium Deoxycolate plus protease and phosphatase inhibitors. After centrifugation at 14 000 g for 20 min at 4 °C, supernatants were quantified for protein concentration (Bradford, Biorad), mixed with SDS-PAGE sample buffer, boiled, and subjected to 8–15% SDS-PAGE. Gels transferred onto PVDF membranes and processed for specific immunodetection using ECL reagent. For comparative analysis using western-blot, 10 human cases per condition 4 mice per condition were analysed. GAPDH and β-actin antibodies were used for normalization.

### RNA purification and retrotranscription

RNA from different human and mouse brain regions was purified using miRVANA RNA isolation kit following manufacturer’s protocol. RNAs were treated with DNase Set (Qiagen) for 15min to eliminate genomic DNA contamination. The concentration of each sample was measured using a NanoDrop 2000 spectrophotometer (Thermo Scientific). RNA integrity was assessed with the RNA Integrity Number (RIN value) determined with the Agilent 2100 Bioanalyzer (Agilent). The retrotranscriptase reaction of the RNA samples was carried out with the High Capacity cDNA Archive kit (Applied Biosystems).

### RNA-sequencing

The analysis of RNA-seq data was performed as described previously [43]. In brief, RNA-seq data was subjected to an in-house quality control workflow. Read quality was assessed using FastQC (http://www.bioinformatics.babraham.ac.uk/projects/fastqc/) (v0.10.1) to identify sequencing cycles with low average quality, adapter contamination, or repetitive sequences from PCR amplification. Alignment quality was analyzed using samtools flagstat [58] (v0.1.18) with default parameters. RNA-seq data was aligned to the genome using gapped alignment as RNA transcripts are subject to splicing and reads might therefore span two distant exons. Reads were aligned to the whole Mus musculus mm10 genome using STAR aligner [23] (2.3.0e_r291) with default options, generating mapping files (BAM format). Read counts for all genes and all exons (Ensembl annotation v72) were obtained using FeaturesCount (http://bioinf.wehi.edu.au/featureCounts/). For data visualisation, BAM files were converted into WIG and BigWig files using the MEDIPS ‘MEDIPS.exportWIG’ function with a window of 50bp and RPM normalization. For the differential expression analysis, read counts that were generated with FeaturesCount were compared between groups using DESeq2 [64]. Genes with a >0.5 logFC cut off and FDR-adjusted *p*-value smaller or equal to 0.05 were considered to be differentially expressed.

### qPCR

Quantitative RT-PCR assays were performed in duplicate on cDNA samples in a LightCycler® 480 System from Roche. The reactions were carried out using 20xTaqMan Gene Expression Assays (Additional file 1: Table S1) and 2xTaqMan Universal PCR Master Mix (Applied Biosystems). The reactions were conducted following the parameters: 50°C for 2 min, 95°C for 10 min, 40 cycles at 95°C for 15 s and 60°C for 1 min. The fold change was determined using the equation 2-ΔΔCT. Mean fold-change values were analysed with appropriate statistical test indicated in each figure using GraphPad Prism 6.01.

### Immunohistochemistry and immunofluorescence

Immunohistochemical study was performed on 4 μm-thick dewaxed paraffin sections of control and sCJD cases. Tissue sections were boiled in citrate buffer for 20 min to retrieve antigenicity. Endogenous peroxidases were blocked with peroxidase (Dako) followed by 10% normal goat serum. Following incubation with the primary antibody at room temperature overnight, the sections were incubated with EnVision + system peroxidase (Dako) at room temperature for 15 min. The peroxidise reaction was visualized with diaminobenzidine (DAB) and H2O2. The omission of the primary antibody in some sections was used as a control for the immunostaining; no signal was obtained with the incubation only of the secondary antibody. No immunogenic peptides were available for any antibody used. Sections were slightly counterstained with haematoxylin.

Immunofluorescence for CamKIIβ, S100A6, Calpain1 and 2 and CHOP was carried out on de-waxed sections, 4 μm-thick, which were stained with a saturated solution of Sudan black B (Merck, DE) for 15 min to block the autofluorescence of lipofuscin granules present in cell bodies, and then rinsed in 70% ethanol and washed in distilled water. The sections were boiled in citrate buffer to enhance antigenicity and blocked for 30 min at room temperature with 10% foetal bovine serum diluted in PBS. Then, the sections were incubated at 4°C overnight with combinations of primary antibodies. After washing, the sections were incubated with Alexa488 or Alexa546 (Molecular Probes, US) fluorescence secondary antibodies against the corresponding host species. Nuclei were stained with DRAQ5™ (1:2.000, Biostatus, UK). After washing, the sections were mounted on Immuno-Fluore mounting medium (ICN Biomedicals, US), sealed, and dried overnight. Sections were examined with a Leica TCS-SL confocal microscope. Again, omission of the primary antibody in some sections was used as a control for the immunostaining.

Double immunofluorescence for Cathepsin S, SIM32, LAMP2, CD68 and HLA-DR were performed in 4% formalin fixed and paraffin-embedded tissues from the cerebellum of human sCJD. The 4μm-thick dewaxed sections were treated for 60 min. with pH 6.0 citrate solution (Dako, DK) for antigen retrieval. The sections were incubated at 4 °C overnight with combinations of primary antibodies. As primary antibodies, anti-CD68 (1:50), anti-cathepsinS (1:50), anti-LAMP2 (1:50), anti- phosphorylated neurofilaments (1:1000), anti-neurofilament H non-phosphorylated (1:50), anti-HLA-DR, (1:20) were used. The fluorescence-labelled secondary antibodies were Alexa Fluor 488 (donkey anti-mouse, 1:200, Molecular Probes, USA by Life Technologies) and Cy5 (goat anti-rabbit, 1:100, Jackson ImmunoResearch, US). The following combinations of antibodies were applied: CathepsinS (Cy5)/CD68 (AF488), CathepsinS (Cy5)/LAMP2 (AF488), CathepsinS (Cy5)/SMI 31 (AF488), CathepsinS (Cy5)/SMI32 (AF488), and CathepsinS (Cy5)/HLA DRAF488). Tissue slides were mounted with Prolong Gold antifade reagent with DAPI (Molecular Probes by Life Technologies, US). The omission of the primary antibody was used as a control for the immunostaining.

The staining results were evaluated with a confocal laser-scanning microscope (FV1200, Olympus, Japan). Image sizes of 1024 ~ 1024 pixels were obtained to allow for the greatest spatial discrimination between pixels and maximise resolution potential in the XY dimension.

### Transmission electron microscopy

For electron microscopy, approximately 2-mm3 samples of grey matter from sCJD prefrontal cortex were prepared. Wherever possible, at least two grids were prepared from every block. The tissue blocks were immersion-fixed in 2.5% glutaraldehyde for less than 24 h, embedded in Epon and routinely processed for electron microscopy. Grids were examined and photographed in JEOL JEM 100 CX and JEOL JEM 1011 transmission electron microscopes at 80 kV. Analysis of fibril diameter was performed using iTEM Soft Imaging System for transmission electron microscopy, Olympus, Japan.

### Immunoprecipitation

Immunoprecipitation experiments were performed by using magnetic Dynabeads with protein G (Life Technologies) according to manufacturer’s instructions with few modifications. Briefly, Dynabeads were equilibrated with 0.3% CHAPS and antibody coupled for 30 min at 4 °C with slight rotation. 500 μg of tissue lysate was added to the antibody-coupled Dynbeads and incubated for overnight at 4 °C with slight rotation. Dynabead complexes were washed three times with 0.3% CHAPS each for 1 min. Elution was performed by resuspending complexes in sample loading buffer (2X) and boiling at 95 °C for 5 min.

### Aggregation assays

Recombinant chimeric PrP (2 μg), which consists of the Syrian hamster PrP (residues 14 to 128) followed by sheep PrP (residues 141 to 234 of the R154 Q171 polymorphic haplotype) was incubated with 0.1% w/v brain cell lysates from control and sCJD brains in the presence or absence of proteases inhibitors cocktail (Roche) or MDL28170. Reactions were performed at 30°C O/N with soft shaking (150 rpm). Samples were either mixed with LB (2x) and analysed by western-blot against PrP (SAF70) antibody or centrifuged at 14000 rpm for 15 min at room temperature. Supernatant (soluble) and pellet (insoluble) fractions were quantified using Bradford method. Four frontal cortex cases were analysed per condition.

### Solubility assay and subcellular fractionation

Solubility assays were performed as previously described [32] with minor modifications. Brain samples (30mg) from control and sCJD MM1 cases were homogenized in a Polytron homogenizer (full speed) in 750 μL of ice-cold PBS+ (sodium phosphate buffer pH 7.0, plus protease inhibitors) and centrifuged at 5.200 rpm at 4°C for 10-min. The pellet was discarded and the resulting supernatant was centrifuged at 16500 g at 4°C for 90 min. The supernatant (S2) was kept as the PBS-soluble fraction. The resulting pellet was re-suspended in a solution of PBS, pH 7.0, containing 0.5% sodium deoxycholate, 1% Triton, and 0.1% SDS, and this was centrifuged at 16500 g at 4°C for 90 min. The resulting supernatant (S3) was kept as the deoxycholate-soluble fraction. The corresponding pellet was re-suspended in a solution of 2% SDS in PBS with occasional mixing and maintained at room temperature for 30-min. Immediately afterward, the samples were centrifuged at 16500 g at 25°C for 90 min and the resulting supernatant (S4) was the SDS-soluble fraction. Fractions were analysed by Western-blot for PrP and Calpain-1 antibodies as described above.

Lysosome-enriched fraction and cytoplasmic fraction were prepared from prion protein peptide treated and untreated primary cell cultures. Samples were homogenized in 0.25 M sucrose, 10 mM HEPES (pH 7.4), 100 mM EDTA, and centrifuged at 6500g, 4°C, for 5 min. The supernatant was further centrifuged at 15000 g, 4°C for 20 min. The pellet (enriched lysosomal fraction) and the supernatant (cytoplasmic fraction) were mixed with SDS-PAGE sample buffer.

### RT-QuIC

The RT-QuIC was performed as described previously [21, 81] with minor modifications. Recombinant PrP was seeded with clarified 10% w/v brain homogenates lysed in PBS 0.1% SDS and diluted 10–8 in PBS in a 96-well black optical bottom plate (Fisher-Scientific). Each sample was run in duplicate. Prepared plates were sealed (VWR) and incubated in a FLUO Star OPTIMA plate reader (BMG Labtech) at 42°C for 80 h with intermittent shaking cycles, consisting of one minute double orbital shaking at the highest speed (600 rpm) followed by a 1-min break. Beta-sheet formation kinetics was determined by measuring the Thioflavin-T (ThT) fluorescence signal (450 nm excitation and 480 nm emission) every 30 min in relative fluorescence units (rfu). In vitro proteolytic assays with active human Calpain 1 (Millipore) were performed on 1% (w/v) brain lysates for 30 min at 37°C in buffers recommended by commercial suppliers.

### Statistical analysis

Pearson r and statistical significance (*p* value) was calculated to indicate correlations between different groups of samples. The ANOVA was followed by a Tukey’s Multiple Comparison post-hoc test when values from different groups were compared. Unpaired two-tailed *t*-test was used when two groups of samples where compared. GraphPad Prism 6.01 was used for statistical calculations. Differences between groups were considered statistically significant at * *p* < 0.05, ** *p* < 0.01, and *** *p* < 0.001.

## Results

### Altered Ca^2+^ homeostasis and ER stress in sCJD brain

To identify differentially expressed genes during development of sCJD pathology we analysed the expression levels in the cortical region of tg340-*PRNP*129MM mice infected with sCJD MM1 brain homogenates at pre-clinical (120 dpi) and clinical (180 dpi) stages and compared with those obtained from control infected animals. These mice are an excellent model of sCJD pathogenesis since they fully recapitulate the neuropathological and molecular features of sCJD MM1 subtype cases [15, 62, 70].

**Fig. 1:**
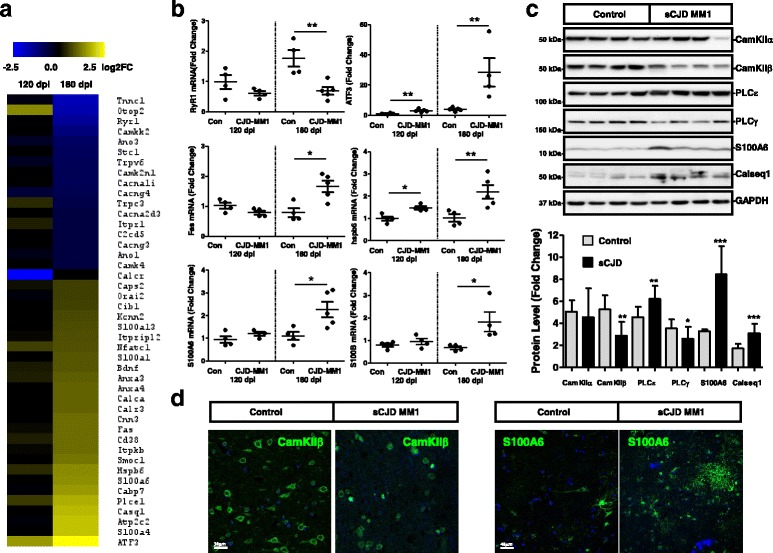
Altered Ca^2+^ homeostasis in sCJD. **a** Heat map analysis of regulated Ca^2+^ related genes in the cortical region of the tg340-*PRNP*129MM sCJD mice at 120 (pre-clinical) and 180 (clinical) days after inoculation with sCJD MM1 brain homogenates compared with control inoculated mice. Data were generated by RNA-sequencing analysis as indicated in material and Methods section. Regulated genes were considered those with log2FC superior or equal to 0.5 and *p* value <0.05. **b** qPCR validation of selected genes involved in Ca^2+^–dependent cellular responses at 120 dpi and 180 dpi in the sCJD infected tg340 mice. Four to five animals were analysed per time point and condition. **c** Western-blot (*n* = 14/group) and (**d**) immunohistochemistry validation in human sCJD MM1 cases from selected proteins regulated in the sCJD infected tg340 mice. Unpaired *t*-test (95% CI) was used for the comparisons of the two groups. *p < 0.05; **p < 0.01; ***p < 0.001

Analysis of RNA-sequencing indicated a massive deregulation of Ca^2+^ related genes in sCJD infected mice, especially at clinical stages (Fig. 1a). Among them, we detected Ca^2+^ binding proteins, Ca^2+^ channels and Ca^2+^-dependent cellular responses, suggesting an alteration of Ca^2+^ homeostasis. Selected mouse genes falling into these categories were validated by qPCR: downregulation of the Ca^2+^release channel Ryanodine receptor 1 (RYR-1), and upregulation of the Ca^2+^-binding proteins S100 Ca^2+^-binding protein (S100)B and S100A6, the heat shock protein family B (Small) member 6 (hspb6/hsp20), the Ca^2+^-responsive Fas gene and the nuclear Ca^2+^ signalling target AMP-dependent transcription factor (ATF)3, were confirmed (Fig. 1b).

Next we validated at protein level in sCJD the alteration on Ca^2+^-response proteins previously detected in the RNA-seq analysis (Fig. 1c and d). Downregulation of Phospholipase C (PLC)γ and Ca^2+^/calmodulin-dependent protein kinase II (CamKII)β, but not of CamKIIα, and upregulation of PLCε, and S100A6 proteins was detected by western-blot in sCJD cases compared to controls (Fig. 1c). Lower levels of CamKIIβ in neurons and increased S100A6 in reactive astroglia and neurons in sCJD were further detected by immunohistochemistry analysis (Fig. 1d).

Since a link between ER stress responses and Ca^2+^ homeostasis has been suggested in prion diseases [91] we next analysed activation state of the UPR, which is activated in prion disease models [46, 68]. No significant differences were observed in the levels of CCAAT-enhancer-binding protein homologous protein (CHOP/GADD153) between control and sCJD cases in human and mice (Additional file 2: Figure S1A, B and C). No alterations in the cleavage of ATF6 from the full length (p90) to the cleaved form (p50) were observed. However, significant increase in the IRE-1 phosphorylation was detected in sCJD without a concomitant increased expression of total IRE-1 (Additional file 2: Figure S1A). Partial UPR response coupled to the upregulation of the UPR/ER related proteins grp78, hsp27 and Bcl-2 and the Ca^2+^ responsive proteins BDNF and Fas in sCJD without alteration of Bcl-2/Bax ratio (Additional file 3: Figure S2A and B) indicates the existence of a complex stress-related cascade of Ca^2+^-dependent responses in human prion pathogenesis.

### Deregulated expression and activation of Calpains in sCJD

**Fig. 2:**
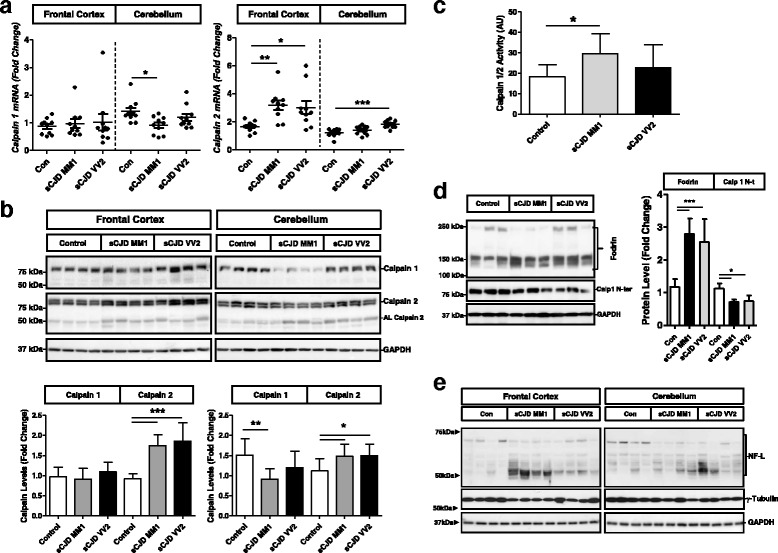
Regional and subtype-dependent expression and activation of Calpains in sCJD. **a** qPCR analysis of expression levels of Calpain 1 and Calpain 2 in the frontal cortex and cerebellum of control, sCJD MM1 and sCJD VV2 cases. **b** Western-blot and densitometry of Calpain 1 and Calpain 2 in the frontal cortex and cerebellum of control, sCJD MM1 and sCJD VV2 cases (*n* = 14/group). **c** Calpain activity by means of fluorometric assay based on the detection of cleavage of Calpain substrate Ac-LLY-AFC in the frontal cortex of control, sCJD MM1 and sCJD VV2 cases (*n* = 6/group). **d** Western-blot and densitometric analysis of Fodrin and N-terminal cleaved Calpain-1 in the frontal cortex of control, sCJD MM1 and sCJD VV2 cases (*n* = 6/group). ANOVA test followed by post-test Tukey’s Multiple Comparison Test was used to compare the values from different groups. P values for the comparisons of the three groups are indicated in the figure:*p < 0.05; **p < 0.01; ***p < 0.001

Increase in intracellular Ca^2+^ concentration leads to the activation of the Ca^2+^-dependent non-lysosomal cysteine proteases Calpains, which are over activated in several pathological conditions including neurodegenerative diseases [26, 47]. Thus, Calpain expression and activation state was analysed in sCJD. At mRNA expression level, Calpain 2 (CAPN2) was overexpressed in the frontal cortex and cerebellum of sCJD MM1 and VV2 subtypes. In spite of a slight downregulation of Calpain 1 (CAPN1) levels in the cerebellum of sCJD MM1 cases, no major alterations were detected for Calpain 1 (Fig. 2a) and for the small regulatory Calpain subunit 4 (CAPN4/CAPSN1) (Additional file 4: Figure S3A). Analysis at protein level matched data obtained at mRNA level. In addition, the presence of autolytic Calpain 2 bands was found in sCJD cases as an indirect observation of increased Calpain activity (Fig. 2b). Fluorescent enzymatic activity assays demonstrated an increase of Calpain 1/2 activity in the frontal cortex of sCJD MM1 cases compared to controls. Increased Calpain activity was also observed in sCJD VV2 cases compared to controls, but without reaching statistical significance (Fig. 2c). Decreased Calpain 1 levels detected with an antibody for the N-terminal region that is cleaved on its activation process (Fig. 2d) support Calpain activation in sCJD brain. Additionally, sCJD cases presented increased Fodrin (Fig. 2d) and Neurofilament Light (NFL) cleavage and decreased γ-tubulin levels, both known cellular endogenous Calpain substrates (Fig. 2e and Additional file 4: Figure S3B).

**Fig. 3:**
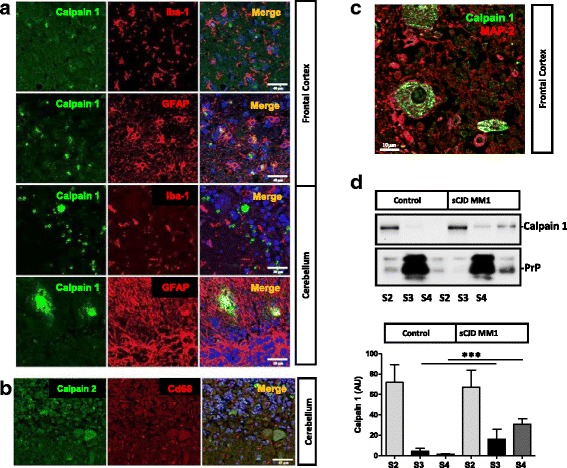
Neuronal localization of Calpains in sCJD. **a** Immunohistochemical staining of frontal cortex and cerebellum sCJD stained either with Calpain 1 (green) and Iba-1 or GFAP (red). Tissues were counterstained with DAPI (blue). **b** Immunohistochemical staining of cerebellum sCJD stained with Calpain 2 (green) and Cd68 (red). Tissues were counterstained with DAPI (blue). **c** Immunohistochemical staining of frontal cortex sCJD stained with Calpain 1 (green) and MAP2 (red). Calpains are mainly expressed in neurons as shown in the merged panels. **d** Western-blot analysis of solubility assays in control (*n* = 3) and sCJD MM1 cases (*n* = 3) by means of differential centrifugation in which cytoplasm fraction (S2), membrane fraction (S3) and insoluble fraction (S4) were obtained. Samples were developed for Calpain1 and PrP antibodies. . Unpaired *t*-test (95% CI) was used for the comparisons of the two groups. *p < 0.05; **p < 0.01; ***p < 0.001

In order to determine the neural cell type expression and subcellular localization of Calpains in sCJD brains, double immunohistochemistry analysis with neuronal and glial markers as well as solubility assays were performed. Calpain expression in CD68+ and GFAP+ cells was residual both in the frontal cortex and cerebellum regions of sCJD cases (Fig. 3a and b). On the contrary, Calpain 1 expression was predominant in MAP2+ cells (Fig. 3c). In control cases, Calpain 1 was localized in the cytoplasmic fraction (S2); while PrP was mainly present in membrane fractions (S3), despite being detectable in the cytoplasmic (S2) and SDS-soluble fractions (S4) (Fig. 3d). In sCJD, Calpain 1 levels were increased in the S3 and S4 fractions in agreement with Calpain activation at the cell membrane following interaction with membrane bound phospholipids [25]. As expected, increased PrP levels in sCJD were detected in the SDS soluble fractions as a consequence of its increased aggregation and insolubility on its pathogenic form (Fig. 3d).

### Impairment of lysosomal integrity and autophagy function in sCJD

**Fig. 4:**
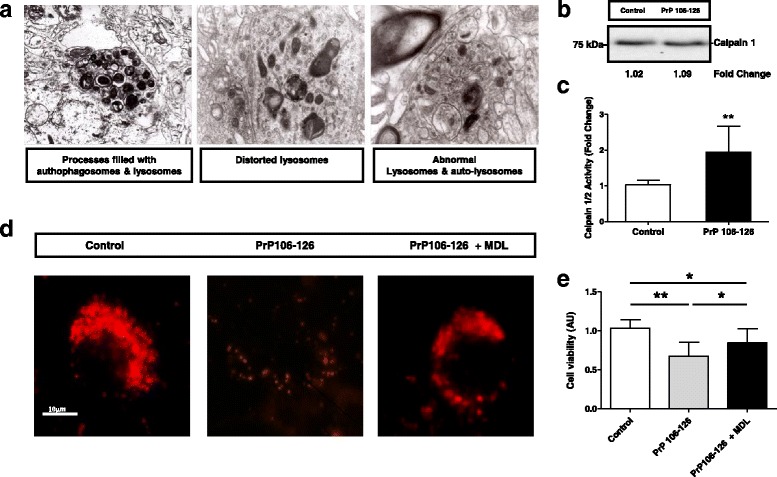
Abnormal lysosomes in sCJD are dependent on Calpain over activation. **a** TEM images indicating the presence of processes with autophagosomes and lysosomes, distorted and abnormal lysosomes and auto-lysosomes in neurons of the frontal cortex of sCJD cases. **b** and **c** Increased Calpain 1/2 activity by fluorimetric Calpain activity (**c**) without alterations on Calpain 1 levels (**b**) in PCC treated with the prion protein peptide (106–126). Inhibition of Calpain activity by MDL28170 treatment partially reverses (**d**) decrease on Lysotracker signal and decrease on cell viability (**e**) caused by prion protein peptide treatment. Unpaired *t*-test (95% CI) was used for the comparisons of the two groups. Data from PCC are obtained from three independent experiments. ANOVA test followed by post-test Tukey’s Multiple Comparison Test was used to compare the values from different groups. P values for the comparisons of the three groups are indicated in the figure:*p < 0.05; **p < 0.01; ***p < 0.001

**Fig. 5:**
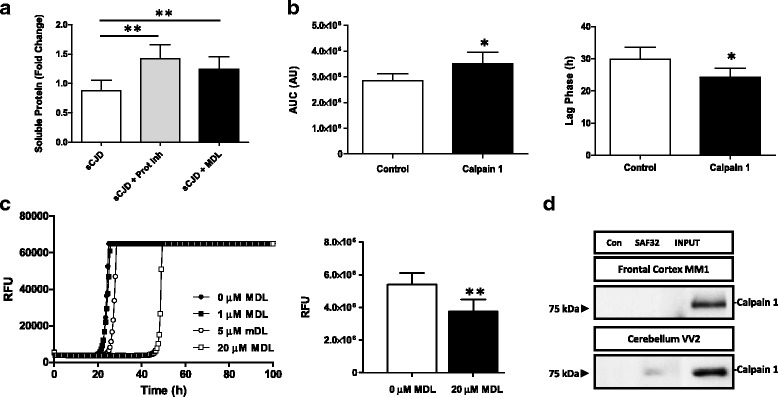
Calpain-dependent PrP solubility and prion conversion in sCJD. **a** Solubility assay in sCJD brain homogenates (*n* = 3) in presence of recombinant PrP (rPrP) and in the presence or absence of protein inhibitors and the Calpain inhibitor MDL28170. Quantification of soluble rPrP is shown. **b** RT-QuIC assay performed in the presence of sCJD brain homogenates (*n* = 4) as seeding material, previously treated or untreated with recombinant Calpain. Area under the curve (AUC) (left) and Lag Phase (hours) are shown. **c** RT-QuIC assay from tg340-*PRNP*129MM brain extracts (*n* = 3) inoculated with sCJD MM1 previously incubated with increased concentrations of MDL28170. Relative Fluorescence Units (RFU) are shown (**d**) Co-immunoprecipitation study of Calpain 1 and PrP in the frontal cortex of sCJD MM1 and in the cerebellum of sCJD VV2 cases. Calpain 1 antibody was used for western-blot immunodetection. Control indicates the use a non-specific antibody as immunoprecipitating antibody. Unpaired *t*-test (95% CI) was used for the comparisons of the two groups. ANOVA test followed by post-test Tukey’s Multiple Comparison Test was used to compare the values from different groups. *P* values for the comparisons of the three groups are indicated in the figure:*p < 0.05; **p < 0.01; ***p < 0.001

The pathogenic activation of Calpain in the intracellular compartment leads to a broad range of degenerative conditions in the brain [96]. Among them, Calpains induce lysosomal permeabilization and disruption as a consequence of the unspecific cleavage of membrane proteins [88, 97]. The presence of neuronal processes filled with autophagosomes and lysosomes, distorted lysosomes, abnormal lysosomes and auto-lysosomes was a general feature in the analysis of sCJD cases by Transmission electron microscopy (TEM) (Fig. 4a). This was accompanied by the expression of autophagy related genes in the tg340-*PRNP*129MM -sCJD mice (Additional file 5: Fig. S4A and B) and in sCJD cases (Additional file 5: Figure S4C and D). The increase in LC3 II, DJ-1 as well as of the heat shock proteins HSPA8 and HSPB8 detectable in sCJD, along with decreased ATG5 and unaltered LAMP2 levels (Additional file 5: Figure S4C and 4D) suggested that, although autophagy mechanisms may be switched in sCJD, this process is not fully functional, in agreement with the presence of abnormal autophagy vesicles (Fig. 5a).

To test the role of Calpain activation in lysosomal disruption, primary cortical neurons (PCC) were treated with the aggregated prion protein peptide 106–126, an ex vivo model of prion toxicity leading to synaptic damage and neurotoxicity [40, 67]. Prion protein peptide treatment did not alter Calpain 1 expression (Fig. 4b) but induced an increase in Calpain activity; resembling observations in sCJD (Fig. 4c). In addition, prion protein peptide treatment impaired lysotracker signal suggesting lysosomal dysfunction in peptide-treated neurons, an effect that was partially reverted by the addition of the Calpain inhibitor MDL28170 (Fig. 4d). Accordingly, MDL28170 also significantly protected neurons from prion protein peptide mediated toxicity (Fig. 4e).

### Calpain activity modulates Prion conversion

In addition to the well-known neurotoxic effects of Calpain over activation in pathological conditions, Calpain has been described to cleave pathological PrP forms and Calpain inhibition prevents the accumulation of PrP^Sc^ in prion models [101]. Thus, activation of Calpain in sCJD cases prompted us to speculate a potential role of Calpain in the PrPSc mediated pathogenesis.

Incubation of recombinant PrP (rPrP) with sCJD brain extracts showed an increase in the formation of SDS-PAGE resistant oligomeric rPrP forms compared to incubation with control brain extracts (Additional file 6: Fig. S5). To demonstrate the role of proteases in PrP aggregation, solubility assays were performed with rPrP incubated with sCJD extracts in the presence and absence of protease inhibitors and MDL28170. Both treatments reduced the amount of insoluble rPrP formed upon its incubation with sCJD extracts (Fig. 5a).

In order to analyse if exacerbated proteolytic activities were altering not only PrP aggregation but also its seeding ability, brain extracts were incubated with recombinant Calpain. Resulting reactions were used as a seeding material for real-time quaking-induced conversion (RT-QuIC) assays, an vitro amplification technology for detection of the abnormal form of prion protein (PrP^Sc^) and the quantification of its prion-seeding activity [5, 21, 66] as well as for the characterization of compounds inhibiting or enhancing PrP conversion [81].

Increased fluorimetric signal as measured by the quantification of the resulting Area Under the Curve (AUC) and decreased lag phase indicated that Calpain activity in sCJD lysates enhanced PrP seeding ability over rPrP compared to non-treated lysates (Fig. 5b). Additionally, pre-treatment of tg340-*PRNP*129MM sCJD mice brain extracts with MDL28170 reduced RT-QuIC signal (decreased AUC and increased lag phase), in a dose dependent manner (Fig. 5c). To validate the specificity of pathogenic PrP as seeding agent in the RT-QuIC reactions performed in the presence of Calpain, the anti-prion compound doxycycline (DOX) [81] was added into the assay, which completely inhibited fluorimetric signal (Additional file 7: Fig. S6).

The observation of Calpain accumulations in the cerebellum of sCJD tissue suggested that Calpain may be involved in the physiopathological mechanisms of PrP aggregation forming stable protein-protein complexes, not limited to an enzyme-substrate interaction. Immunoprecipitation experiments demonstrated that Calpain 1 and PrP do not interact in the frontal cortex of sCJD, and only slight signal was detected in the cerebellum region, ruling out the possibility of a functional interaction between both proteins (Fig. 5d).

### Cathepsin S activation in sCJD

**Fig. 6:**
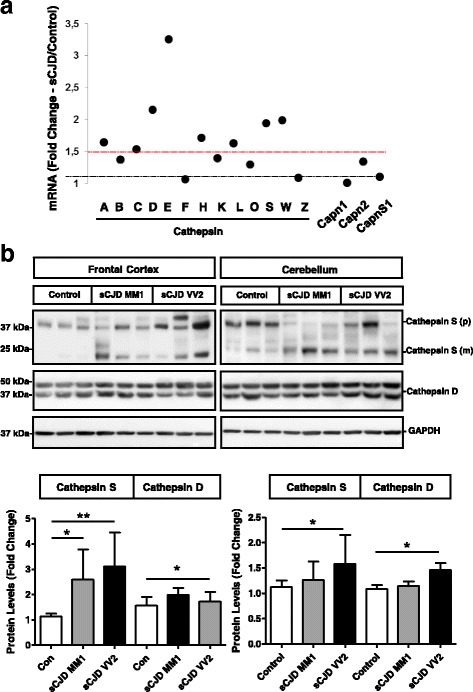
Altered Calpain levels in sCJD. **a** Expression of Cathepsin family, Calpain 1 (Capn1), Calpain 2 (Capn2) and Calpain 4 (Capns1) in the cortical region of the tg340-*PRNP*129MM mouse model at 180 (clinical) days after inoculation with sCJD MM1 brain homogenates. Data were generated by RNA-sequencing analysis. Fold Change line at 1.5 indicates the threshold of significant regulations in the expression levels for these genes between control and sCJD MM1 inoculated mice. **b** Western-blot and densitometry analysis of Cathepsin S and Cathepsin D expression in the frontal cortex and cerebellum of control (*n* = 9), sCJD MM1 (*n* = 9) and sCJD VV2 (*n* = 9) cases. ANOVA test followed by post-test Tukey’s Multiple Comparison Test was used to compare the values from different groups. *P* values for the comparisons of the three groups are indicated in the figure:*p < 0.05; **p < 0.01

**Fig. 7:**
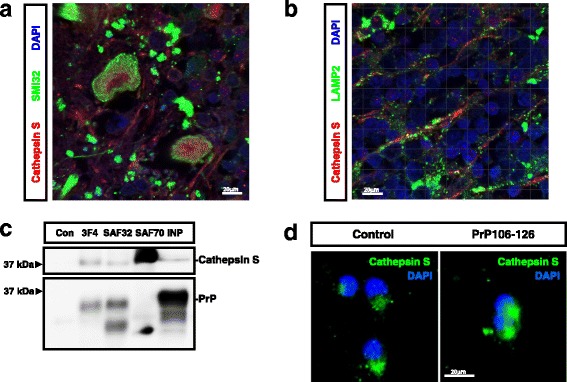
Neuronal Cathepsin S in sCJD. Immunohistochemical staining of FC sCJD cases double-immunostained with Cathepsin S (red) and (**a**) SIM32 (green) or LAMP2 (**b**). Tissues were counterstained with DAPI (blue). **c** Co-Immunoprecipitation study of Cathepsin S and PrP in the frontal cortex of sCJD cases. Three different anti-PrP antibodies recognizing independent epitopes were used for Immunoprecipitation (3F4, SAF32 and SAF70). Western-blots were developed with a Cathepsin S antibody. Control indicates the use a non-specific antibody as immunoprecipitating antibody. **d** Immunohistochemistry images of Cathepsins S (green) in PCC treated or untreated with the prion peptide. Cells were counterstained with DAPI

The demonstration of neuronal lysosomal disruption in sCJD made us speculate that release of lysosomal content, especially Cathepsins, could be a key determinant on the neurotoxicity linked to prion diseases through the so-called Calpain-Cathepsin hypothesis observed in some neurological and neurodegenerative conditions [88, 103]. We profiled the complete Cathepsin family signature in the cortex of the tg340-*PRNP*129MM sCJD mice from the RNA-seq dataset at clinical stages. Eight members of the Cathepsin family as well as their endogenous inhibitor Cystatin C appeared overexpressed compared to controls (Fig. 6a). Among upregulated Cathepsins, Cathepsin S retaining proteolytic activity at neutral pH [53], and Cathepsin D, associated with increased risk of variant CJD [10] and with enhanced immunoreactivity in sCJD [56], were initially explored. In addition, both genes were previously reported to be upregulated in scrapie infected mice [22, 100]. Overexpression of Cathepsin S, especially in the frontal cortex, as well as the presence of mature (active) Cathepsin S bands, was detected in sCJD irrespective of the brain region and disease subtype, in agreement with our previous observation of increased Cathepsin S mRNA in sCJD [62]. Moderate increase in Cathepsin D was detected in the frontal cortex of sCJD MM1 cases and in the cerebellum of sCJD VV2 cases (Fig. 6b). Neuronal Cathepsin S was principally localized in the axons, although some staining in the soma was also detectable (Fig. 7a). Only partial overlap between Cathepsin S and the lysosomal marker LAMP2 staining was detected (Fig. 7b) indicating that Cathepsin S is mainly located outside the lysosomal compartment in sCJD, in agreement with the presence of lysosomal damage (Fig. 4a). Although it was technically not possible to detect PrP-Cathepsin S colocalization in sCJD neurons, three different PrP antibodies did immunoprecipitate Cathepsin S from sCJD brain extracts (Fig. 7c). Additionally, in PCC Cathepsin S localized in granule-like cytoplasmic regions, while treatment with the prion protein peptide induced changes in the immunofluorescence signalling to a diffuse staining pattern in the neuronal soma suggesting Cathepsin S release from intracellular organelles (Fig. 7d). Accordingly, lysosomal enriched fractions derived from prion protein peptide treatments presented a decrease in Cathepsin S content, while a slight increase in cytoplasmic fraction could be detected (Additional file 8: Figure S7).

**Fig. 8:**
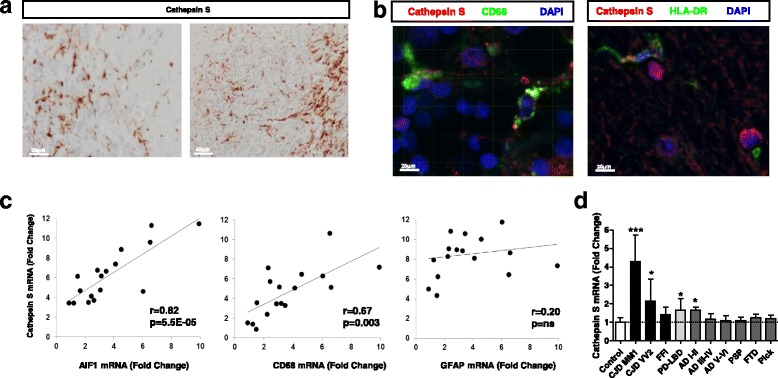
Microglial overexpression of Cathepsin S in sCJD. **a** Immunohistochemical analysis of Cathepsin S expression in cerebellum of sCJD cases showing microglial localization. **b** Immunohistochemical staining of sCJD cases in the frontal cortex double-immunostained with Cathepsin S (red) and CD68 (green) left and Cathepsin S (red) and HLA-DR (green) right. Tissues were counterstained with DAPI (blue). **c** Correlations between the levels of Cathepsin S and glial markers (AIF1 and CD68 for microglia and GFAP for astroglia) in the frontal cortex of sCJD cases. R and p values (Pearson correlation) are indicated. **d** Expression levels of Cathepsin S in the frontal cortex of several neurodegenerative diseases with known cortical affection by means of qPCR analysis FFI: Fatal Familial Insomnia, PD-LBD: Parkinson Disease-Lewy Body Dementia, AD: Alzheimer’s Disease, Braak Stages I-II and III-IV, PSP: Progressive supranuclear palsy, FTD: Frontotemporal dementia, Pick: Pick’s disease. *P* values for the comparisons of the disease groups with control cases are indicated in the figure:*p < 0.05; **p < 0.01; ***p < 0.001

Our immunohistochemical analysis revealed no major alterations in neuronal Cathepsin S levels in sCJD brain tissue and prion protein peptide treated PCC, which stands in clear contrast with elevated total expression levels by qPCR and western-blot. Since Cathepsin S is involved in a broad range of inflammatory-related pathological stimuli [20], the presence of a secondary role for Cathepsin S in glial cells of sCJD was investigated. Immunohistochemistry analysis revealed a major Cathepsin S microglial staining in sCJD (Fig. 8a). These observations were validated in double Cathepsin S-CD68 and Cathepsin S-HLA-DR immunostainings (Fig. 8b) and further confirmed by the finding that Cathepsin S mRNA levels correlated with those of microglial markers (AIF1 and CD68), while no correlation was observed with the astrocytic marker GFAP (Fig. 8c). The analysis of the Cathepsin S expression levels by qPCR in the frontal cortex of several neurodegenerative diseases with cortical affection indicated that Cathepsin S sCJD overexpression is not a common feature of neurodegenerative diseases, although modest increases on its expression was also detected in Parkinson Disease/Lewy Body Dementia (PD/LBD) and in early stages of AD (Fig. 8d).

### Activation of Calpain-Cathepsin axis is an early event in sCJD pathogenesis

**Fig. 9:**
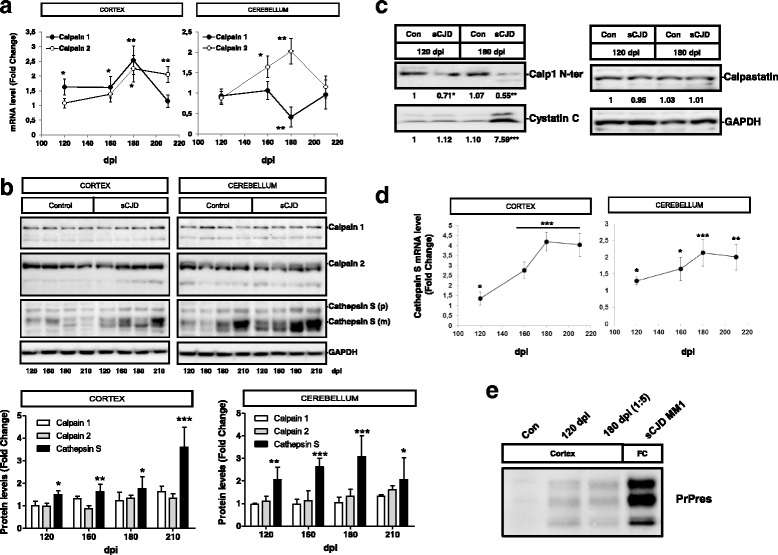
Activation of Calpain-Cathepsin in sCJD at pre-clinical stages of the disease. **a** qPCR analysis of expression levels of Calpain 1 and Calpain 2 in the cortex and cerebellum of the tg340-*PRNP*129MM mice inoculated with at sCJD MM1 at 120, 160, 180 and 210 dpi (10–1 inoculum dilution was used for animals sacrificed at 210 dpi). Fold Change represents the ratio between animals inoculated with sCJD-MM1 and control homogenates. **b** Western-blot and densitometry of Calpain 1, Calpain 2and Cathepsin S in the cortex and cerebellum of control (*n* = 3) and sCJD MM1 (*n* = 3) inoculated tg340-*PRNP*129MM mice at sCJD MM1 at 120, 160, 180 and 210 dpi (10–1 inoculum dilution was used for in animals sacrificed at 210 dpi). Fold Change represents the ratio between animals inoculated with sCJD-MM1and control homogenates. **c** Western-blot analysis of Calpain 1 N-ter, Cystatin C and Calpastatin in the cortex and cerebellum of control (*n* = 3) and sCJD MM1 (*n* = 3) inoculated tg340-*PRNP*129MM mice at pre-clinical (120dpi) and clinical (180dpi) stages. Fold change between sCJD MM1 and control inoculated animals is indicated. **d** qPCR analysis of expression levels of Cathepsin S in the cortex and cerebellum of the tg340-PRNP129MM mice inoculated with at sCJD MM1 at 120, 160, 180 and 210 dpi (10–1 inoculum dilution was used for animals sacrificed at 210 dpi). Fold Change represents the ratio between animals inoculated with sCJD-MM1 and control homogenates. **e** Western-blot analysis of PK-treated brain extracts from tg340-*PRNP*129MM mice inoculated with control and sCJD MM1 bran homogenates at 120 and 180 dpi. 180 dpi sample was diluted 1:5. ANOVA test followed by post-test Tukey’s Multiple Comparison Test was used to compare the values from different groups. *P* values for the comparisons of the three groups are indicated in the figure:*p < 0.05; **p < 0.01; ***p < 0.001

Time-dependent alterations of Calpain-Cathepsin axis in sCJD pathogenesis were analysed in the tg340-*PRNP*129MM sCJD mice. Increased mRNA Calpain levels were detected at differential disease stages with exception of a decrease of cerebellar Calpain 1 levels at 180 dpi (Fig. 9a). However, alterations at mRNA levels were not translated into major changes at protein level, besides a slight increase in the expression of autolytic bands in sCJD infected animals (Fig. 9b) in agreement with observations in human samples. Decreased levels of Calpain 1, detected by a Calpain N-terminal directed antibody indicated the presence of active Calpain at pre-clinical and especially at clinical stages of the disease. Analysis of endogenous Calpain inhibitors expression revealed the presence of increased Cystatin C at clinical stages of the disease, but unaltered Calpastatin levels (Fig. 9c).

Increase in Cathepsin S mRNA and protein was detected at pre-clinical sCJD stages, and more significantly, at clinical stages (Fig. 9d). Importantly, the presence of cleaved Cathepsin S mature bands was already present at pre-clinical sCJD stages (Fig. 9b). Alterations in Calpain and Cathepsin expression levels and their activation at pre-clinical stages correlate with the presence of pathogenic PrP, in form of Proteinase K-resistant PrP (PrPres), whose levels are already detectable at pre-clinical stages but in lower amounts (5 times lower) than at clinical stages (Fig. 9e).

All together indicates that Calpain and Cathepsin S activation are parallel events during development of sCJD and that Calpain-Cathepsin axis activation is an early event in disease pathogenesis.

## Discussion

As a consequence of the conformational changes in PrP^C^ leading to the formation and accumulation of pathological PrP forms (PrP^Sc^), multiple mechanisms operate in a concerted manner promoting the spread of the disease throughout the brain and the manifestation of the prion-related pathology. The nature of the primary contributors to neurodegeneration in prion infected neurons is unclear, since many molecular mechanisms and cellular pathways are simultaneously altered and acting interconnected in a synergic manner [54]. In addition, initial neuroprotective events, such as neuroinflammation, may become toxic after pathological threshold has been reached [1].

Plasma and ER membrane channel receptors and intracellular Ca^2+^ sensors play a key role in maintaining physiological Ca^2+^ concentrations in the cytoplasm. When Ca^2+^ homeostasis is unbalanced, sustained increase in cytoplasmic Ca^2+^ is a common initial step of irreversible injury in neurons [35]. The presence of altered Ca^2+^ homeostasis has been suggested in prion models [91] although experimental evidence of its occurrence in human prion diseases was not reported so far. In sCJD brain tissue we detected massive alterations in the expression levels of Ca^2+^-dependent genes, including Ca^2+^ binding proteins, plasma membrane and ER Ca^2+^ receptors and Ca^2+^ signalling genes. While these regulations were mainly detectable at clinical stages of the disease, alterations in the expression of several Ca^2+^-related genes were also found at pre-clinical stages, when accumulation of pathological PrP in form of PrPres was also detected. This is in agreement with recent data suggesting that disturbed Ca^2+^ homeostasis and Ca^2+^-mediated signalling is a common feature in early stages of several neurodegenerative diseases such as PD and AD [48, 50, 87, 99]. Additionally, in AD, disrupted neuronal Ca^2+^ homeostasis exacerbates Aβ formation and promotes tau hyper-phosphorylation [9].

The primary reason of altered Ca^2+^ homeostasis in sCJD is not clear, but accumulation of misfolded PrP and consequent malfunction of protein quality control machinery could lead to deregulation of intracellular Ca^2+^ [90, 91]. Several mechanisms can contribute to increased Ca^2+^ influx from the extracellular space: i) the presence of reactive oxygen species; as a consequence of oxidative stress [24], a main hallmark in prion pathogenesis [11, 29], ii) loss of PrP^C^ function in the plasma membrane, leading to an impairment of the neuroprotective role of PrP^C^ as modulator of glutamate receptors [14, 52] and iii) the presence of soluble PrP amyloid oligomers binding to cellular receptors leading to disruption to the cell membrane and formation of pores in the cell membrane leading to calcium influx [16, 51, 85].

Our observations indicate that a pleiade of Ca^2+^-related genes present an altered expression in sCJD. Ca^2+^ binding proteins (i.e.: S100 family members, calsequestrin, smoc1 and cabp7) and Ca^2+^-regulated genes (i.e.: BDNF, Bcl-2 and ATF3) were upregulated in sCJD, while Ca^2+^ and cation channels (i.e.: Cacn family members, RyR1, Itpr1) displayed decreased levels compared to controls. Elevated expression of Ca^2+^ binding proteins may be a neuroprotective response to buffer excess of intracellular Ca^2+^, as it occurs under excitotoxic conditions [78]. Interestingly, regulation of Ca^2+^ related proteins is not restricted to neuronal cells and thus, increased immunoreactivity of Ca^2+^ binding proteins such as S100A6 was also detected in reactive astrocytes where S100A6 upregulation may play a role in glutamate toxicity [102]. Increased S100A6 levels have also been reported in other neurodegenerative diseases such as AD and ALS [12].

Alteration of neuronal Ca^2+^ homeostasis in prion disease models induces the release of stored ER- Ca^2+^ leading to ER stress, which is associated with the upregulation of several ER-chaperones and to an increase of the UPR when subjected to ER-stressors [44, 90]. Indeed, chronic ER stress emerges as a key pathological mechanism in prion pathogenesis, not only for its contribution to neurotoxic mechanisms but also to prion spreading, since Ca^2+^ dependent ER-stress facilitates prion replication [44] and cells expressing familial CJD related PrP-mutants present abnormal Ca^2+^ content and increased susceptible to ER stress-inducing agents than controls [90]. Our study supports the presence of altered Ca^2+^ homeostasis and ER stress together with a partial activation of UPR response in sCJD, being IRE-1 pathway the only UPR contributing branch. This would be in agreement with the previously reported lack of activation of the PERK-eIF2α in sCJD, in contrast to what is observed in AD [94] suggesting the presence of specific ER-stress responses in both diseases. Importantly, IRE-1 has been connected to the autophagy mechanisms that contribute to the eventual apoptotic fate through caspase cascade activation [82] but genetic targeting of its downstream effector XBP-1 did not affect prion replication or pathogenesis [45]. This suggests that the IRE-1/XBP-1 UPR branch, which is activated in human prion diseases as demonstrated in the present work, may not contribute to the occurrence of prion pathology. Interestingly, our study also validates the presence of elevated levels of grp78, a master regulator for ER stress and UPR activation, which has been recently shown to modulate prion propagation in vitro and in vivo [74].

Other consequences of intracellular Ca^2+^ overload are mitochondrial damage, and deregulation of Calpains activity [34, 36, 88]. While decline in mitochondrial machinery has been recently demonstrated in sCJD [4] over activation of Calpains in prion disease has only been suggested in scrapie infected mice [38, 41]. By complementary means we demonstrated the presence of exacerbated neuronal Calpain activity in sCJD brain, which, in turn, mediated lysosome damage or rupture and cell death, both effects partially reversed by a Calpain inhibitor. Additionally, we observed that inhibition of proteolytic activity increases PrP solubility, while Calpain treatment is able to enhance prion conversion by fostering the seeding ability of PrP^Sc^ over physiological PrP, a factor that can contribute to the rapid prion replication process in sCJD brain tissue. Our results are in agreement with the reported proteolytic activity of Calpain over PrP^Sc^. In scrapie infected mice, Calpain inhibition did not only prevent the Calpain-mediated cleavage of PrP^Sc^ and its accumulation, but also increased the disease incubation time [101]. Indeed, multiple lines of evidence support a role for proteolytic PrP^Sc^ cleavage in the neurotropism and phenotypic expression of prion diseases [30, 49, 72].

Additionally, lysosomal damage due to the presence of free radicals derived from oxidative stress and proteolysis of ion channels would turn into depolarization of neurons creating a synergic effect in Ca^2+^ influx in form of a self-perpetuating loop, leading to the pathogenic activation of several mechanisms responding to these insults, such as ER stress, autophagy, oxidative stress and chronic neuroinflammation, which are known mechanisms contributing to prion pathogenesis [28, 29, 46, 59, 62, 84]. Interestingly, our data suggest the presence of activated but impaired autophagy in sCJD, as observed in other neurodegenerative diseases [75], since we detected the accumulation of autophagic vacuoles (autophagosomes or autophagolysosomes), abnormal lysosomes and auto-lysosomes. This would be in agreement with increased LC3-II levels, associated with enhanced autophagosome synthesis or reduced autophagosome turnover, due to delayed trafficking to the lysosomes, or impaired lysosomal proteolytic activity. This may result from an overload of the autophagy system due to the intracellular accumulation of misfolded PrP and lysosomal rupture. Eventually, impaired autophagy will probably impede the clearance of protein aggregates and damaged cell organelles, fuelling oxidative stress mechanisms.

Another consequence of intracellular Ca^2+^ overload and Calpain activation is the pathological deregulation of Cathepsins, and especially of Cathepsin S due to its stability at a neutral or slightly alkaline pH, thus retaining most of its activity outside the lysosome [53]. Our study unveiled a dual neural cell-type specific role for Cathepsin S during prion pathogenesis in neurons and microglial cells. While lysosomal-released neuronal Cathepsin S contributes to prion neurotoxicity, the precise role of overexpressed Cathepsin S in microglia remains to be known. Microglial cells are able to release Cathepsin S into the extracellular space which can remove protein aggregates as a neuroprotective mechanism for PrP^Sc^ clearance [7, 22]. Indeed, our data demonstrates a strong interaction between PrP and Cathepsin S in sCJD brain, suggesting that Cathepsin S could be part of the PrP^Sc^ aggregated complexes. However, Cathepsin S also could play a neurotoxic function inducing neuronal death through unregulated degradation of extracellular matrix proteins [69]. The potential dual role of Cathepsin S in prion pathogenesis may be even more complex since degradation of extracellular matrix proteins is a functional mechanism for cell migration, thus Cathepsin S may assist the migration of microglia [42], potentially enhancing microglia mobilization to cope with PrP^Sc^ accumulation. Of interest, Cathepsin S overexpression is not a general feature of neurodegenerative processes since only slight mRNA upregulation was detected in early AD stages and in PD/LBD cases, thus our work sets an important basis for future studies addressing the true contribution of Cathepsin S in the neuroinflammation processes associated with sCJD pathogenesis.

## Conclusions

**Fig. 10:**
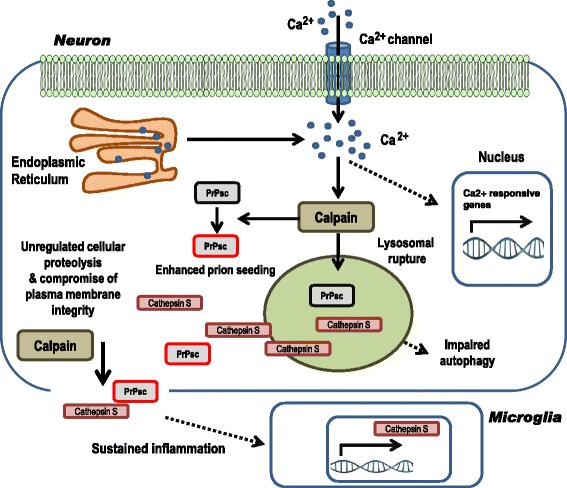
Proposed Calpain-Cathepsin S axis activation in sCJD. As a consequence of increased neuronal intracellular Ca^2+^ concentration in sCJD a broad range of pathologically related events occur such as i) direct or indirect alteration of gene expression patterns and ii) over activation of non-lysosomal cysteine proteases Calpains. On one side, pathological Calpain activity may cleave PrP, enhancing its misfolded conformation and enhancing prion seeding in new conversion cycles. On the other hand, Calpain compromise lysosomal membrane integrity, and as a consequence, Cathepsin proteases are liberated in the cytoplasm. Calpains and proteases with activity at neutral pH, such as Cathepsin S, unspecifically cleave cellular substrates and structures, interfering with physiological cellular functions. When plasma membrane is compromised, the cellular content is released into the extracellular space. Additionally, Cathepsin S expression is overexpressed in microglial cells as a consequence of chronic neuroinflammation

Altogether our study demonstrates that destabilization of neuronal Ca^2+^ homeostasis in sCJD could be one of the upstream and early events leading to the rapid development of the prion disease pathology, in which aberrant Calpain-Cathepsin axis activation would be a key event in the spread and activation of multiple prion disease-related pathological mechanisms (Fig. 10). These findings contribute to a better understanding of molecular mechanisms associated with the development of human prion disease pathogenesis and suggest that Ca^2+^ channel blocking may be a relevant therapeutic strategy as suggested for other neurodegenerative disorders associated with Ca^2+^ alterations such as AD [3, 8, 76]. Importantly, therapeutic intervention would require the definition of very primary causative mechanisms leading to altered Ca^2+^ homeostasis, which in turn unchain the cascade of molecular pathological events, before neuronal damage spreads.

## Additional files

Additional file 1: Table S1. List of Taqman assays used in this study. (PPTX 53 kb)

Additional file 2: Figure S1. Partial UPR activation in the frontal cortex of sCJD cases. (A) Western-blot and densitometric analysis of UPR proteins CHOP, ATF4, P-IRE-1, IRE-1, XBP1 and ATF6 in the frontal cortex of control and sCJD MM1 cases. (B) Immunohistochemical detection of CHOP in the frontal cortex of control and sCJD MM1 cases. (B) Immunohistochemical detection of CHOP in the cortex of control and sCJD MM1 inoculated tg340-*PRNP*129MM mice. Brain slices were counterstained with DAPI. (PPTX 4 kb)

Additional file 3: Figure S2. ER stress in the frontal cortex of sCJD cases. ER stress and Ca^2+^ induced genes in sCJD MM1 by (A) Western-blot (grp78, hsp27, BDNF, Fas and Bcl-2) and (B) Bcl-2/Bax ratio in the frontal cortex of control and sCJD MM1 cases obtained from the densitometric analysis of both proteins detected by western-blot analysis. Unpaired *t*-test (95% CI) was used for the comparisons of the two groups. *p < 0.05; **p < 0.01; ***p < 0.001. (PPTX 130 kb)

Additional file 4: Figure S3. Calpain substrates levels in sCJD. (A) qPCR analysis of CAPNS1/CAPN4 in the frontal cortex and cerebellum of controls and sCJD MM and VV2 cases. (B) Western-blot and densitometry analysis of Neurofilament Light (NF-L) and γ-Tubulin in the frontal cortex and cerebellum of control, sCJD MM1 and sCJD VV2 cases. ANOVA test followed by post-test Tukey’s Multiple Comparison Test was used to compare the values from different groups. P values for the comparisons of the three groups are indicated in the figure:*p < 0.05; **p < 0.01; ***p < 0.001. (PPTX 111 kb)

Additional file 5: Figure S4. Alteration of autophagy related genes in sCJD. (A) Altered expression of genes involved in autophagy in the cortex of the tg340-*PRNP*129MM mice at 120 (pre-clinical) and 180 (clinical) days after inoculation with sCJD MM1 brain homogenates. (B) qPCR analysis of the autophagy activators hspa8 and hspb8 in the sCJD infected tg340-*PRNP*129MM mice at 180 dpi. (C) Western.-blot and densitometric analysis of the autophagy related proteins DJ-1, LC3, ATG5 and LAMP2 in the frontal cortex of control and sCJD MM1 and sCJD VV2 cases. (D) qPCR analysis of the autophagy-related genes HSPA8, HSPB8, PARK (DJ-1) and LAMP2 in the frontal cortex of control and sCJD MM1 and sCJDVV2 cases. Unpaired *t*-test (95% CI) was used for the comparisons of the two groups. ANOVA test followed by post-test Tukey’s Multiple Comparison Test was used to compare the values from different groups. P values for the comparisons of the three groups are indicated in the figure:*p < 0.05; **p < 0.01; ***p < 0.001. (PPTX 244 kb)

Additional file 6: Figure S5. Increased rPrP aggregation induced by sCJD brain homogenates. Recombinant prion protein was incubated with brain homogenates from control and sCJD brains (*n* = 4) and subjected to Western-blot with PrP SAF70 antibody. (PPTX 195 kb)

Additional file 7: Figure S6. Inhibition of RT-QuIC reaction by Doxycycline. RT-QuIC assay performed in the presence of sCJD brain homogenates (*n* = 3) as seeding material, previously treated with recombinant Calpain in the presence (+DOX) of absence (−DOX) of Doxycycline (DOX). Relative Fluorescence Units (RFU) are shown. (PPTX 47 kb)

Additional file 8: Figure S7. Cathepsin S and GAPDH levels in lysosomal and cytoplasmic enriched fractions derived from prion protein peptide treatment. (PPTX 88 kb)

## Abbreviations

AD: Alzheimer’s disease
Ca^2+^: Calcium
ER: Endoplasmic reticulum
*PRNP*: Human prion protein gene
PrP^C^: Cellular prion protein
PrPres: Proteinase-K resistant PrP
PrP^Sc^: Pathogenic scrapie prion protein
rPrP: Recombinant PrP
sCJD: Sporadic Creutzfeldt-Jakob disease
UPR: Unfolded protein response
