# Supplementary figures and images for: Altered Ca2+ homeostasis induces Calpain-Cathepsin axis activation in sporadic Creutzfeldt-Jakob disease
Source: Acta Neuropathol Commun. 2017 Apr 27;5:35. doi: 10.1186/s40478-017-0431-y (PMC5408381; doi:10.1186/s40478-017-0431-y)

## Slide 1
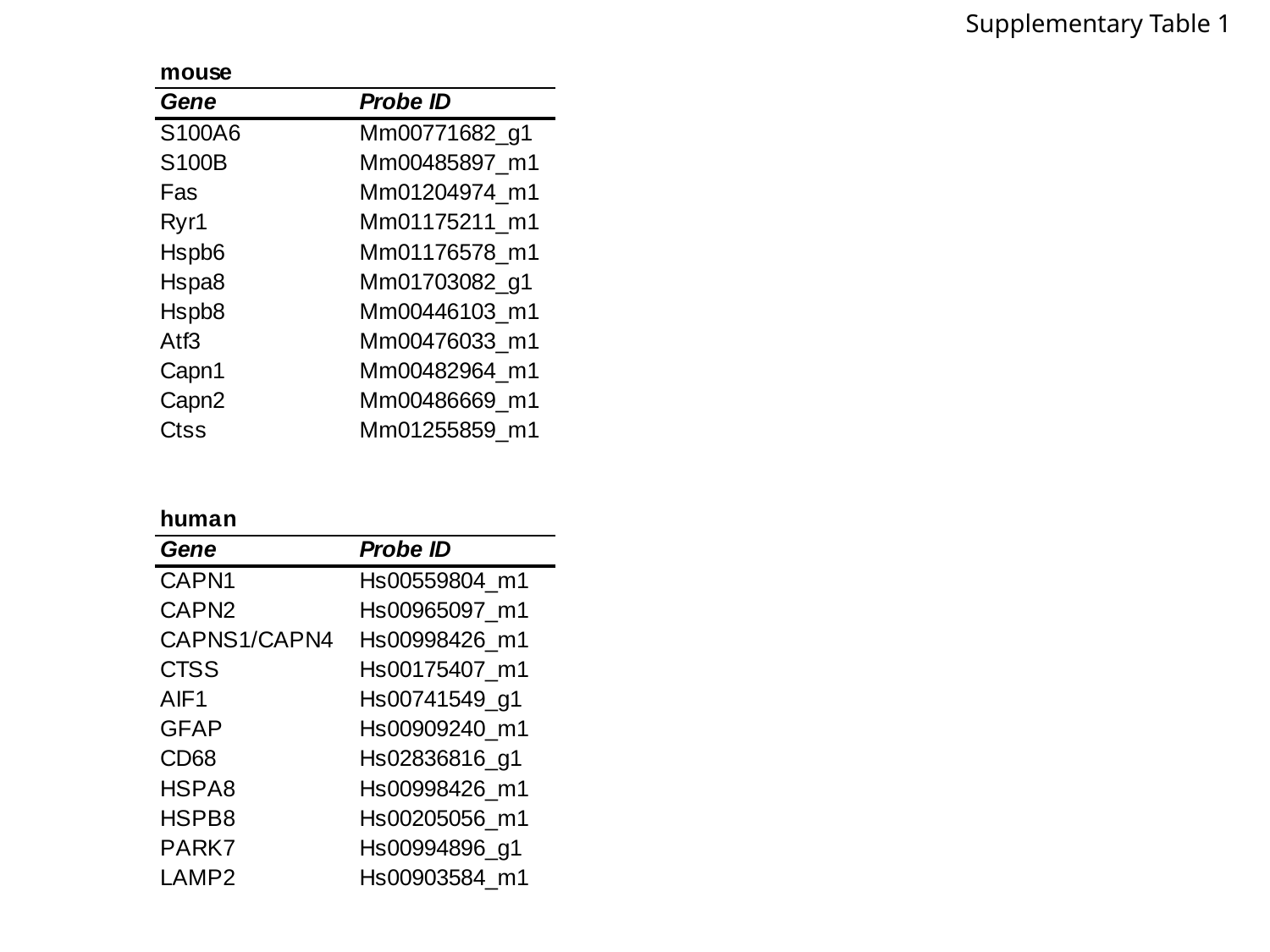

Supplementary Table 1

Supplement: Supplementary file 1 — List of Taqman assays used in this study. (PPTX 53 kb) [file 40478_2017_431_MOESM1_ESM.pptx]

## Slide 1
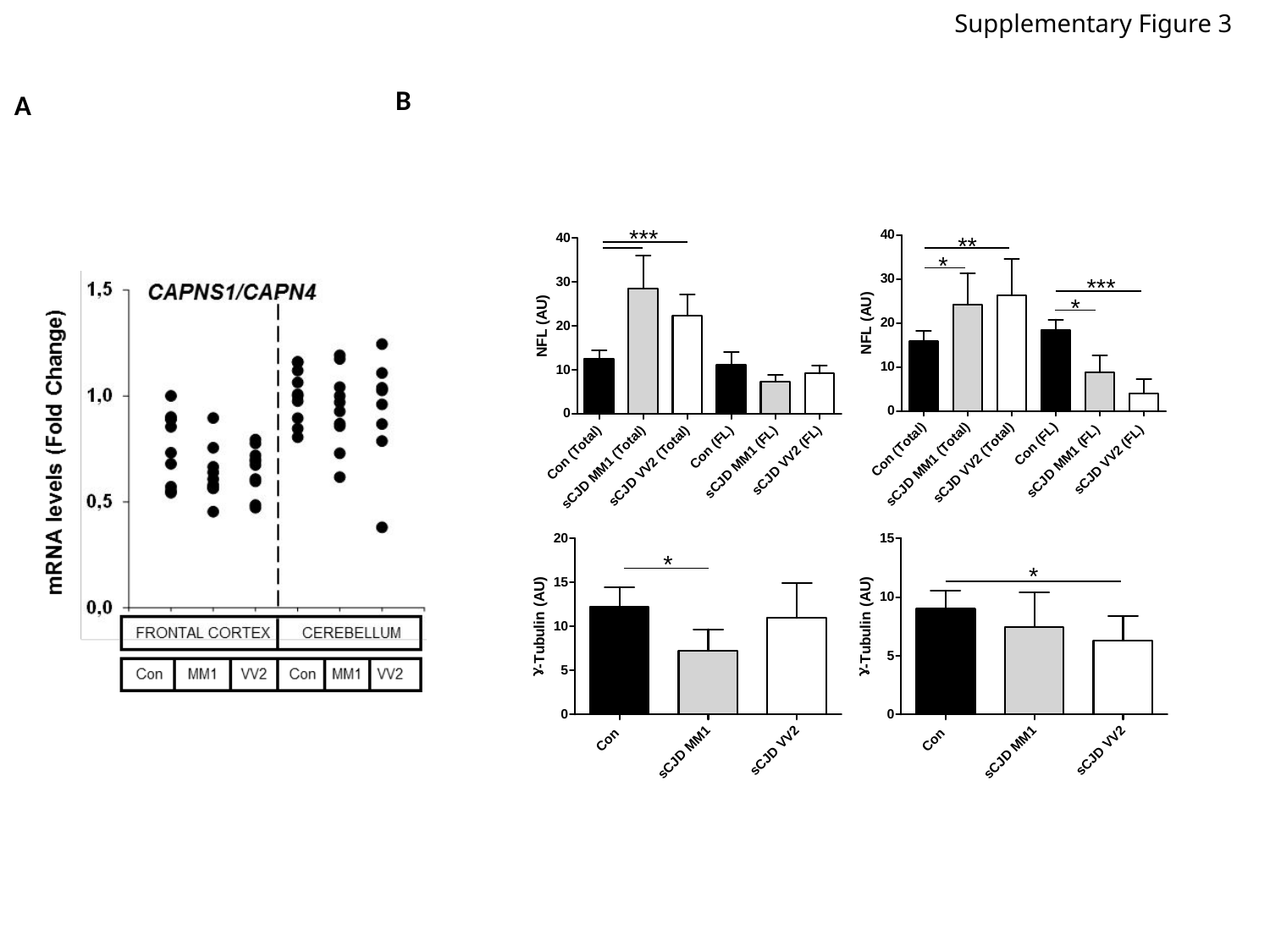

Supplementary Figure 3
B
A
***
**
*
***
*
*
*

Supplement: Supplementary file 4 — Calpain substrates levels in sCJD. (A) qPCR analysis of CAPNS1/CAPN4 in the frontal cortex and cerebellum of controls and sCJD MM and VV2 cases. (B) Western-blot and densitometry analysis of Neurofilament Light (NF-L) and γ-Tubulin in the frontal cortex and cerebellum of control, sCJD MM1 and sCJD VV2 cases. ANOVA test followed by post-test Tukey’s Multiple Comparison Test was used to compare the values from different groups. P values for the comparisons of the three groups are indicated in the figure:*p < 0.05; **p < 0.01; ***p < 0.001. (PPTX 111 kb) [file 40478_2017_431_MOESM4_ESM.pptx]

## Slide 1
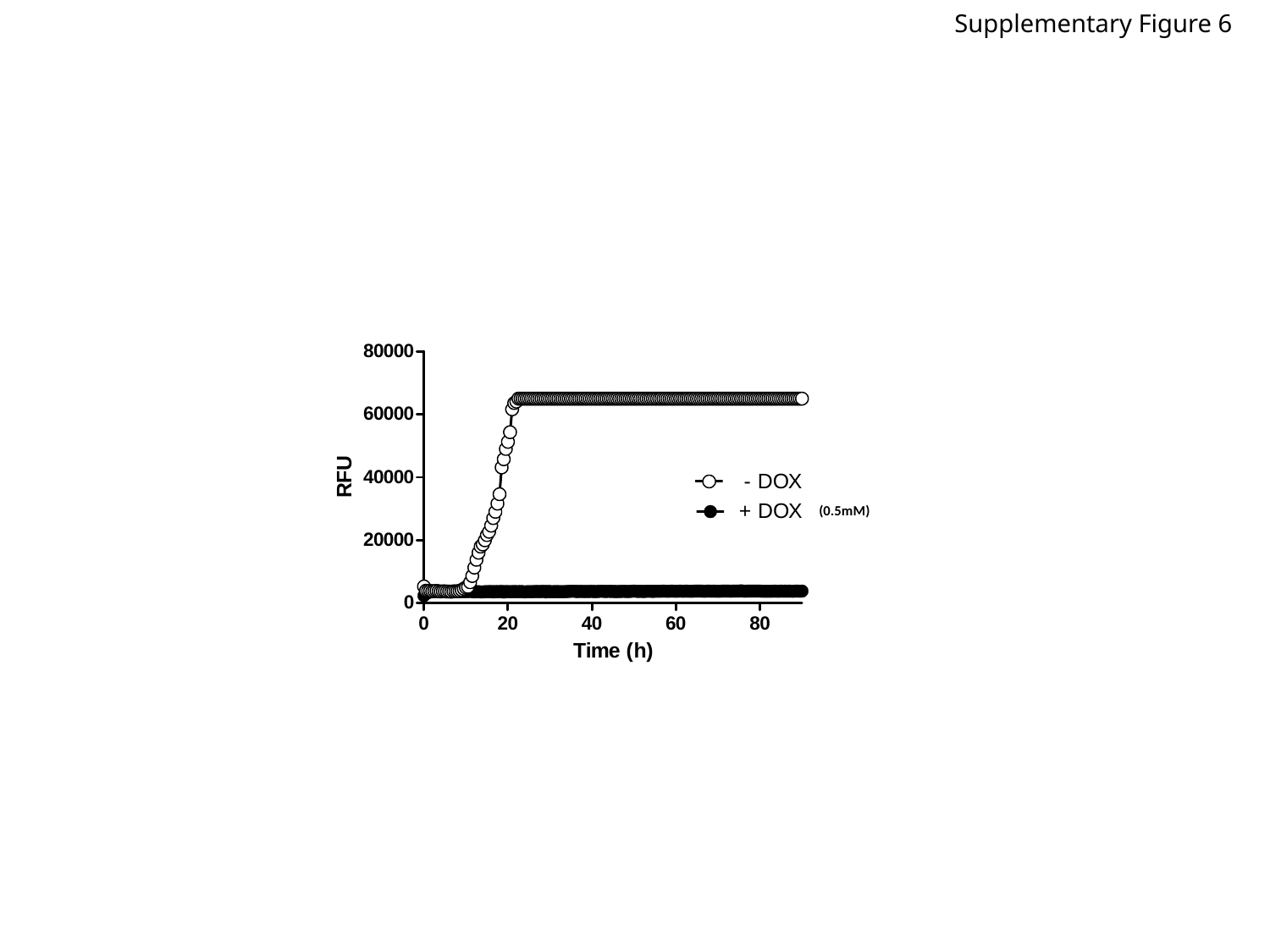

Supplementary Figure 6
(0.5mM)

Supplement: Supplementary file 7 — Inhibition of RT-QuIC reaction by Doxycycline. RT-QuIC assay performed in the presence of sCJD brain homogenates (n = 3) as seeding material, previously treated with recombinant Calpain in the presence (+DOX) of absence (−DOX) of Doxycycline (DOX). Relative Fluorescence Units (RFU) are shown. (PPTX 47 kb) [file 40478_2017_431_MOESM7_ESM.pptx]

## Slide 1
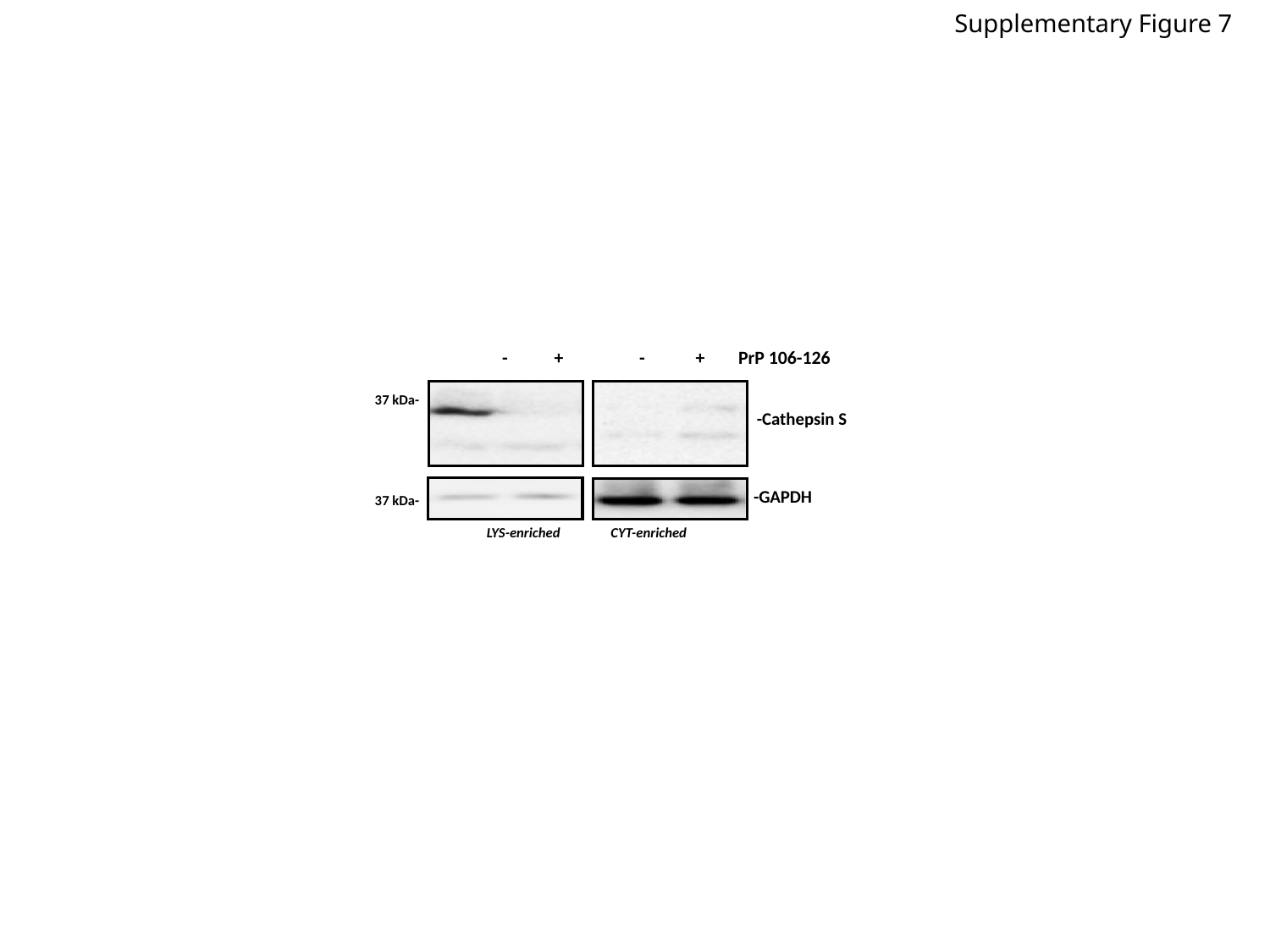

Supplementary Figure 7
 - + - + PrP 106-126
37 kDa-
-Cathepsin S
-GAPDH
37 kDa-
LYS-enriched CYT-enriched

Supplement: Supplementary file 8 — Cathepsin S and GAPDH levels in lysosomal and cytoplasmic enriched fractions derived from prion protein peptide treatment. (PPTX 88 kb) [file 40478_2017_431_MOESM8_ESM.pptx]
