# Supplementary material for: Altered Ca2+ homeostasis induces Calpain-Cathepsin axis activation in sporadic Creutzfeldt-Jakob disease
Source: Acta Neuropathol Commun. 2017 Apr 27;5:35. doi: 10.1186/s40478-017-0431-y (PMC5408381; doi:10.1186/s40478-017-0431-y)
Supplement: Supplementary file 2 — Partial UPR activation in the frontal cortex of sCJD cases. (A) Western-blot and densitometric analysis of UPR proteins CHOP, ATF4, P-IRE-1, IRE-1, XBP1 and ATF6 in the frontal cortex of control and sCJD MM1 cases. (B) Immunohistochemical detection of CHOP in the frontal cortex of control and sCJD MM1 cases. (B) Immunohistochemical detection of CHOP in the cortex of control and sCJD MM1 inoculated tg340-PRNP129MM mice. Brain slices were counterstained with DAPI. (PPTX 4 kb) [file 40478_2017_431_MOESM2_ESM.pptx]

## Slide 1
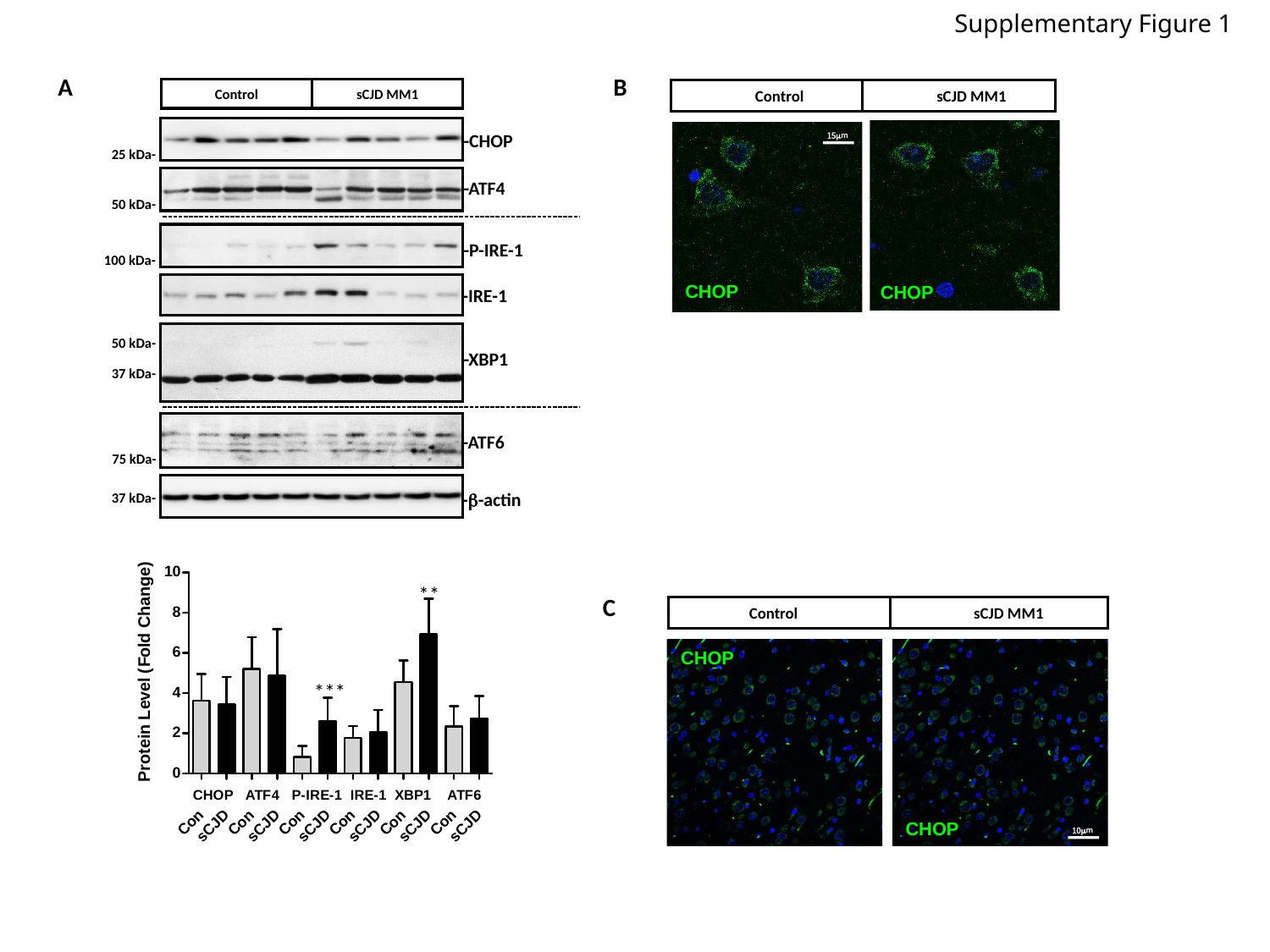

Supplementary Figure 1
A
B
 Control sCJD MM1
 Control sCJD MM1
-CHOP
15mm
25 kDa-
-ATF4
50 kDa-
-P-IRE-1
100 kDa-
CHOP
CHOP
-IRE-1
50 kDa-
-XBP1
37 kDa-
-ATF6
75 kDa-
-b-actin
37 kDa-
**
***
C
 Control sCJD MM1
CHOP
CHOP
10mm
