# Supplementary material for: Altered Ca2+ homeostasis induces Calpain-Cathepsin axis activation in sporadic Creutzfeldt-Jakob disease
Source: Acta Neuropathol Commun. 2017 Apr 27;5:35. doi: 10.1186/s40478-017-0431-y (PMC5408381; doi:10.1186/s40478-017-0431-y)
Supplement: Supplementary file 3 — ER stress in the frontal cortex of sCJD cases. ER stress and Ca2+ induced genes in sCJD MM1 by (A) Western-blot (grp78, hsp27, BDNF, Fas and Bcl-2) and (B) Bcl-2/Bax ratio in the frontal cortex of control and sCJD MM1 cases obtained from the densitometric analysis of both proteins detected by western-blot analysis. Unpaired t-test (95% CI) was used for the comparisons of the two groups. *p < 0.05; **p < 0.01; ***p < 0.001. (PPTX 130 kb) [file 40478_2017_431_MOESM3_ESM.pptx]

## Slide 1
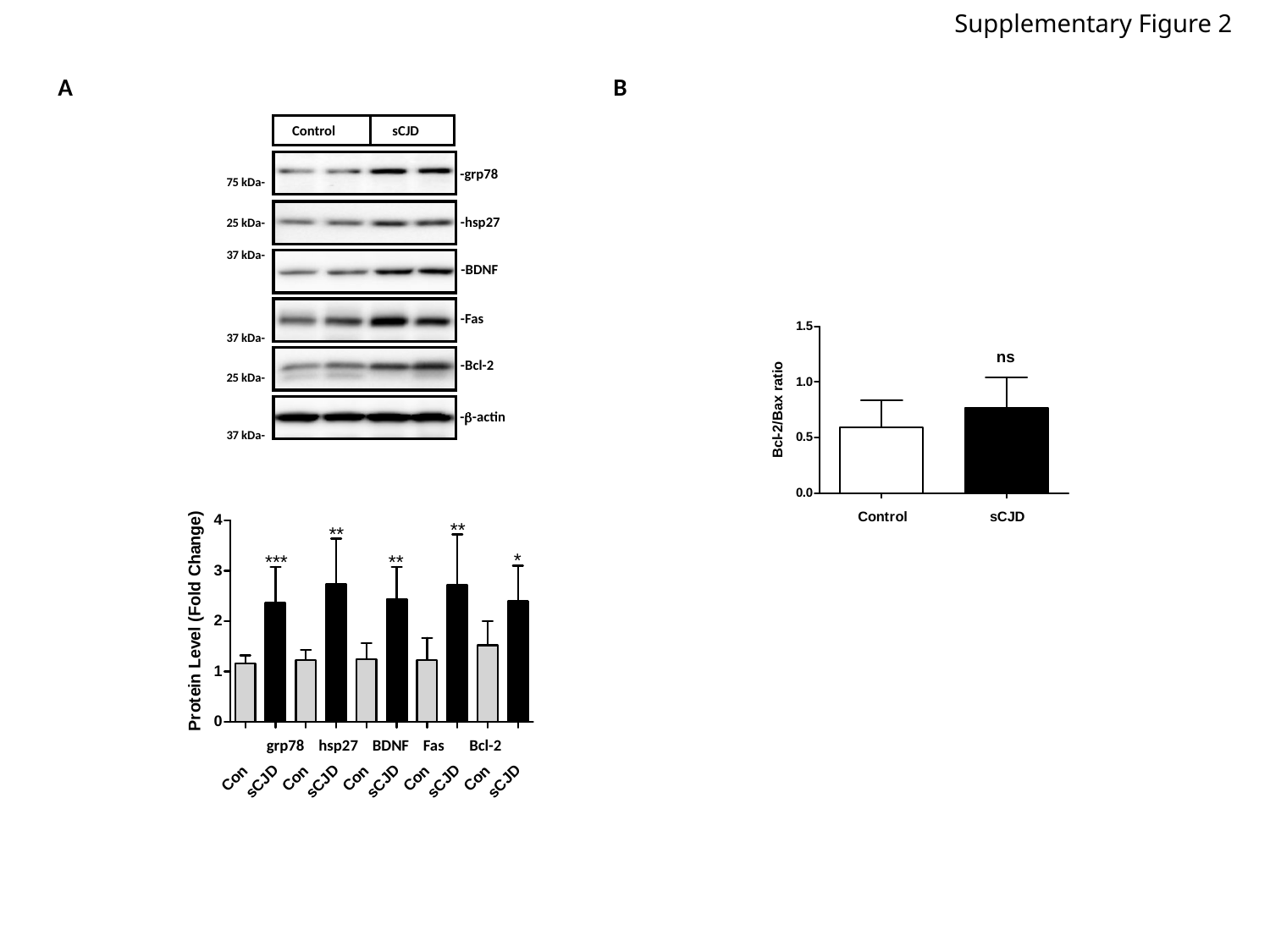

Supplementary Figure 2
A
B
 Control sCJD
-grp78
75 kDa-
-hsp27
25 kDa-
37 kDa-
-BDNF
-Fas
37 kDa-
ns
-Bcl-2
25 kDa-
-b-actin
37 kDa-
**
**
*
***
**
grp78 hsp27 BDNF Fas Bcl-2
