# Supplementary material for: Altered Ca2+ homeostasis induces Calpain-Cathepsin axis activation in sporadic Creutzfeldt-Jakob disease
Source: Acta Neuropathol Commun. 2017 Apr 27;5:35. doi: 10.1186/s40478-017-0431-y (PMC5408381; doi:10.1186/s40478-017-0431-y)
Supplement: Supplementary file 5 — Alteration of autophagy related genes in sCJD. (A) Altered expression of genes involved in autophagy in the cortex of the tg340-PRNP129MM mice at 120 (pre-clinical) and 180 (clinical) days after inoculation with sCJD MM1 brain homogenates. (B) qPCR analysis of the autophagy activators hspa8 and hspb8 in the sCJD infected tg340-PRNP129MM mice at 180 dpi. (C) Western.-blot and densitometric analysis of the autophagy related proteins DJ-1, LC3, ATG5 and LAMP2 in the frontal cortex of control and sCJD MM1 and sCJD VV2 cases. (D) qPCR analysis of the autophagy-related genes HSPA8, HSPB8, PARK (DJ-1) and LAMP2 in the frontal cortex of control and sCJD MM1 and sCJDVV2 cases. Unpaired t-test (95% CI) was used for the comparisons of the two groups. ANOVA test followed by post-test Tukey’s Multiple Comparison Test was used to compare the values from different groups. P values for the comparisons of the three groups are indicated in the figure:*p < 0.05; **p < 0.01; ***p < 0.001. (PPTX 244 kb) [file 40478_2017_431_MOESM5_ESM.pptx]

## Slide 1
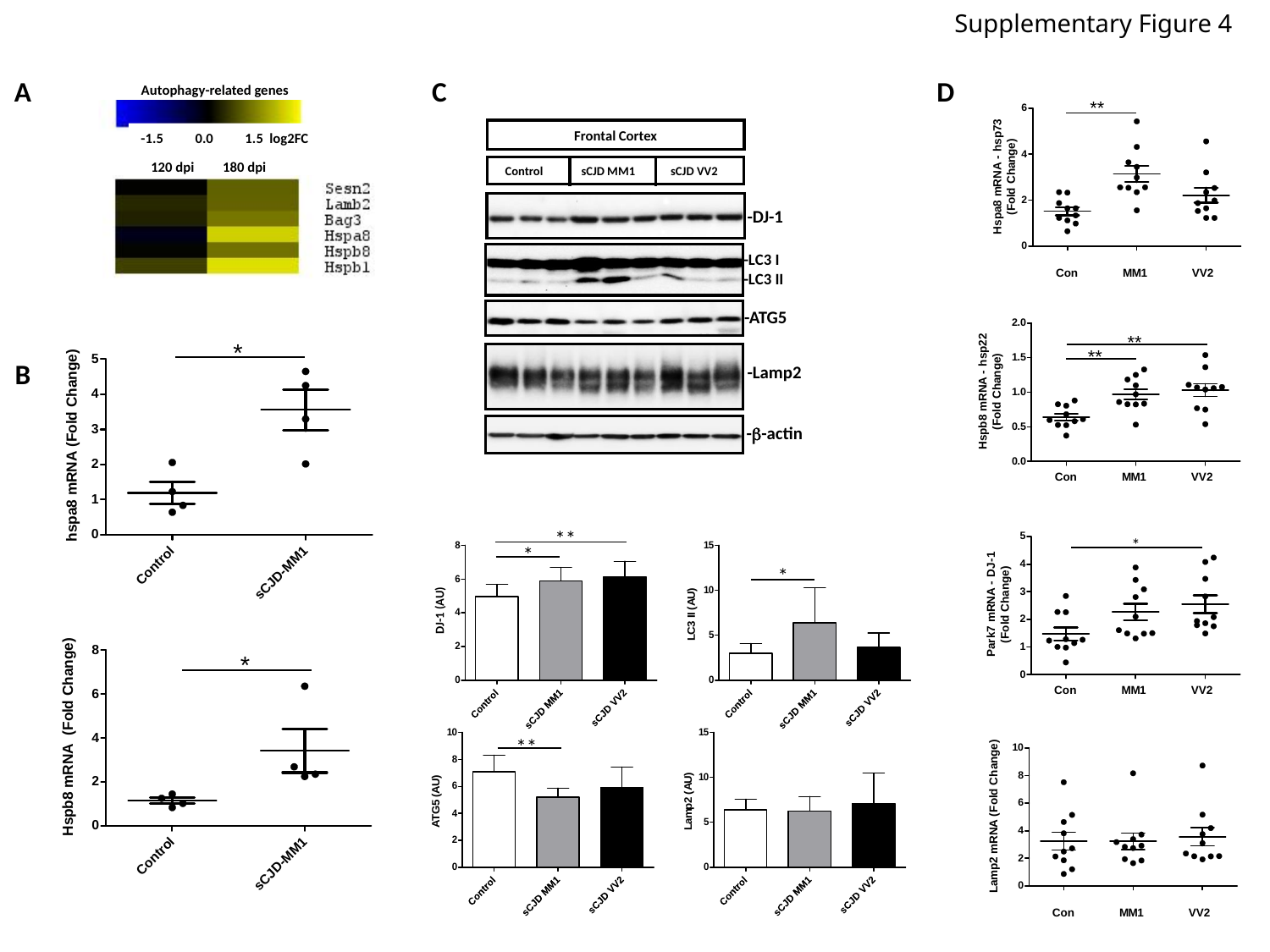

Supplementary Figure 4
A
C
D
Autophagy-related genes
-1.5 0.0 1.5 log2FC
**
Frontal Cortex
120 dpi 180 dpi
 Control sCJD MM1 sCJD VV2
-DJ-1
-LC3 I
-LC3 II
-ATG5
**
*
**
B
-Lamp2
-b-actin
**
*
*
*
DJ-1 (AU)
*
**
