# Supplementary material for: Altered Ca2+ homeostasis induces Calpain-Cathepsin axis activation in sporadic Creutzfeldt-Jakob disease
Source: Acta Neuropathol Commun. 2017 Apr 27;5:35. doi: 10.1186/s40478-017-0431-y (PMC5408381; doi:10.1186/s40478-017-0431-y)
Supplement: Supplementary file 6 — Increased rPrP aggregation induced by sCJD brain homogenates. Recombinant prion protein was incubated with brain homogenates from control and sCJD brains (n = 4) and subjected to Western-blot with PrP SAF70 antibody. (PPTX 195 kb) [file 40478_2017_431_MOESM6_ESM.pptx]

## Slide 1
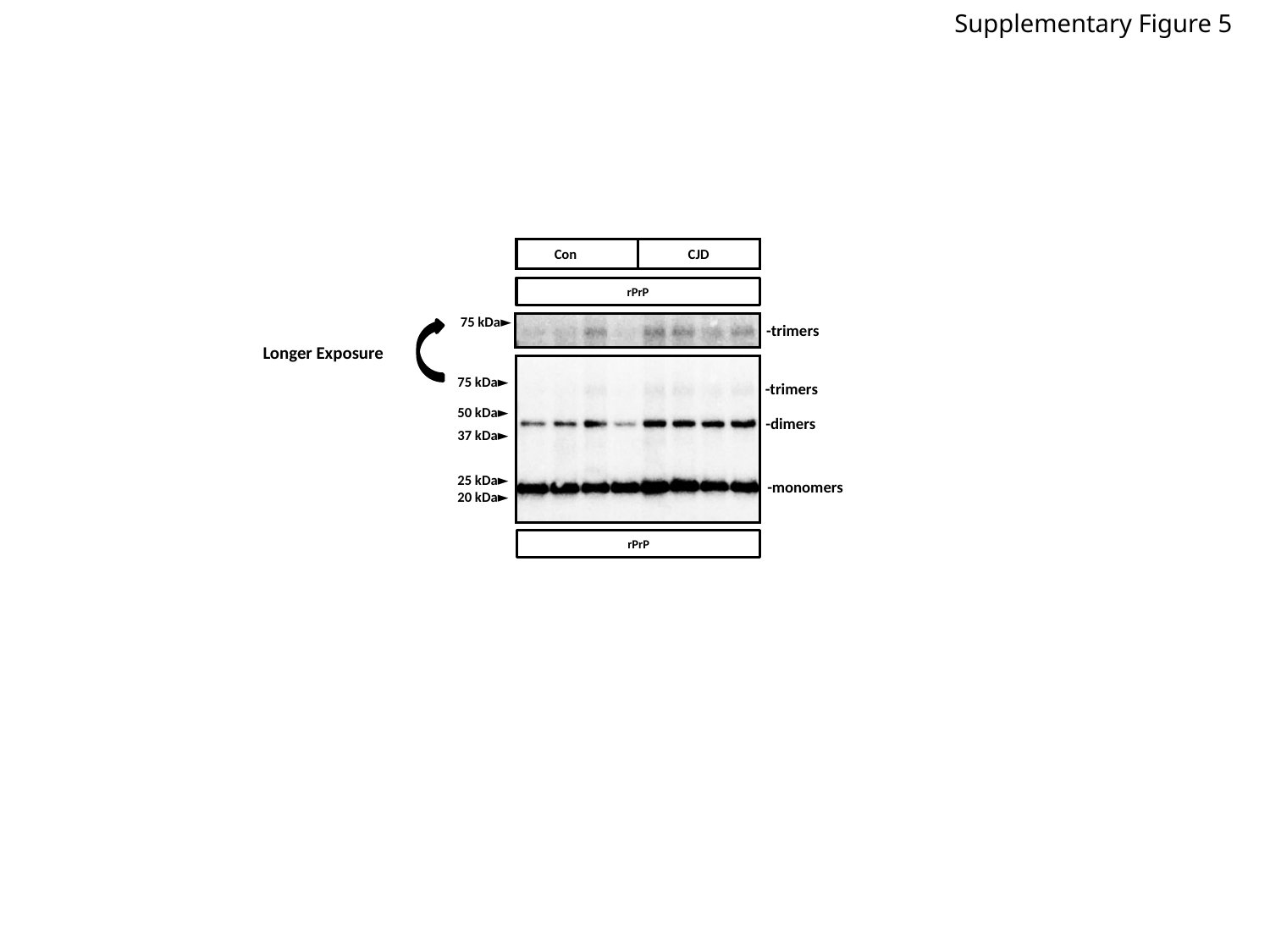

Supplementary Figure 5
 Con CJD
rPrP
75 kDa►
-trimers
Longer Exposure
75 kDa►
-trimers
50 kDa►
-dimers
37 kDa►
25 kDa►
-monomers
20 kDa►
rPrP
